# Experimental Study on Concrete under Combined FRP–Steel Confinement

**DOI:** 10.3390/ma13204467

**Published:** 2020-10-09

**Authors:** Stefan Kaeseberg, Dennis Messerer, Klaus Holschemacher

**Affiliations:** Structural Concrete Institute (IfB), Leipzig University of Applied Sciences, Karl-Liebknecht-Str. 132, 04277 Leipzig, Germany; dennis.messerer@htwk-leipzig.de (D.M.); klaus.holschemacher@htwk-leipzig.de (K.H.)

**Keywords:** reinforced concrete, columns, confinement, CFRP, load-bearing capacity, strengthening

## Abstract

The confinement of reinforced concrete (RC) compression members by fiber-reinforced polymers (FRPs) is an effective measure for the strengthening and retrofitting of existing structures. Thus far, extensive research on the stress–strain behavior and ultimate limit state design of FRP-confined concrete has been conducted, leading to various design models. However, these models are significantly different when compared to one another. In particular, the use of certain empirical efficiency and reduction factors results in various predictions of load-bearing behavior. Furthermore, most experimental programs solely focus on plain concrete specimens or demonstrate insufficient variation in the material properties. Therefore, this paper presents a comprehensive experimental study on plain and reinforced FRP-confined concrete, limited to circular cross sections. The program included 63 carbon FRP (CFRP)-confined plain and 60 CFRP-confined RC specimens with a variation in the geometries and in the applied materials. The analysis showed a significant influence of the compressive strength of the confined concrete on the confinement efficiency in the design methodology, as well as the importance of the proper determination of individual reduction values for different FRP composites. Finally, applicable experimental test results from the literature were included, enabling the development of a modified stress–strain and ultimate condition design model.

## 1. Introduction

**Figure 1:**
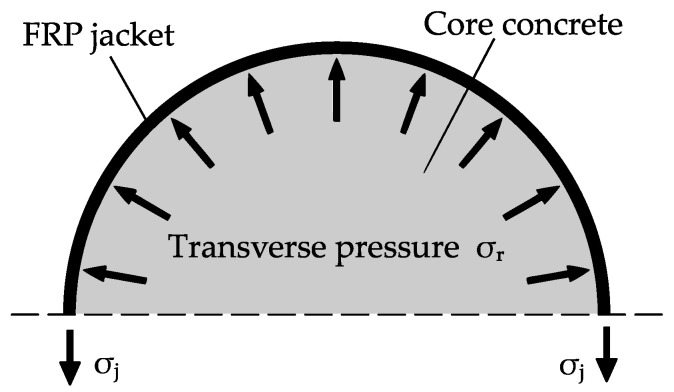
Confining action of a fiber-reinforced polymer (FRP) jacket.

The confinement of axially loaded concrete members is an effective measure for improving load-bearing capacity and ductility. Apart from conventional transverse tie reinforcing steel in combination with shotcrete, fiber-reinforced polymers (FRPs) are becoming increasingly considered for the strengthening and rehabilitation of reinforced concrete (RC) structures. The composite material most commonly combines synthetic fibers (e.g., carbon fibers) and an epoxy-based resin matrix. In the application of confinement, the linear elastic FRP jacket resists the concrete’s lateral expansion, leading to a steadily increasing transverse pressure, *σ*_r_. Regarding circular cross sections, the transverse pressure distributes evenly along the FRP jacket, as shown in Figure 1. The resulting confining pressure is carried by the mostly unidirectionally arranged FRP through tensile stresses *σ*_j_ in the hoop direction. Exceeding the initial compressive strength, an effective confinement leads to a multidimensional stress state of the concrete. Thereby, it is possible to increase its maximum bearing capacity and its ultimate strains without significantly affecting the dead loads. 

The load-bearing behavior of short, plain concrete members confined with FRP composites has been extensively researched in the last two decades, leading to various experimental programs and design models, see, e.g., in [1,2,3,4,5,6,7,8,9,10,11,12,13,14,15,16,17,18,19,20,21,22,23]. To date, these models have already been included in national standards, codes, and guidelines by several countries and institutions, providing frameworks for the design of the FRP confinement of RC columns for strengthening purposes, see, e.g., in [24,25,26,27,28,29,30].

In general, the ultimate confined concrete strength *f*_cc_ and the accompanying axial strain *ε*_ccu_ are derived by Equations (1) and (2):

(1) $$f_{\mathrm{cc}}{\;=\;f}_{c0}{\;+\;k}_{1}\;\cdot {\;f}_{\mathrm{lj}}\;,$$

(2) $$\varepsilon_{\mathrm{ccu}}{\;=\;\varepsilon }_{c0}\;\cdot {\;k}_{2}{\;+\;\varepsilon }_{c0}\;\cdot {\;k}_{3}\;\cdot \;\frac{f_{\mathrm{lj}}}{f_{c0}}\;\cdot \;{\left(\frac{\varepsilon_{\mathrm{ju}}}{\varepsilon_{c0}}\right)}^{k_{4}}\;,$$

where *f*_c0_ is the mean value of the unconfined concrete strength, *ε*_c0_ is the peak strain of the unconfined concrete, *f*_lj_ is the confinement pressure provided by the FRP jacket, *ε*_ju_ is the rupture strain of the FRP jacket in the application of confinement, and *k*_1_–*k*_4_ are factors affecting the impact of *f*_lj_ on *f*_cc_ and *ε*_ccu_. 

The prediction of the ultimate condition of the confined concrete is directly dependent on the confining pressure *f*_lj_ provided by the FRP jacket. The commonly used form for the calculation of the confining pressure is given by Equation (3):

(3) $$f_{\mathrm{lj}}\;=\;\frac{1}{2}\;\cdot {\;\rho }_{j}\;\cdot {\;E}_{j}\;\cdot {\;\varepsilon }_{\mathrm{ju}}{\;=\;E}_{\mathrm{jl}}\;\cdot {\;\varepsilon }_{\mathrm{ju}}\;=\;\frac{2\;\cdot {\;t}_{j}\;\cdot {\;E}_{j}}{D}\;\cdot {\;\varepsilon }_{\mathrm{ju}}\;,$$

where *ρ*_j_ is the confinement ratio, *E*_jl_ is the confinement modulus, *E*_j_ is the modulus of the composite material, *t*_j_ is the FRP thickness, and *D* is the diameter of the circular cross section. 

The rupture strain of the carbon FRP (CFRP) jacket in the application of confinement, *ε*_ju_, has a significant impact on the confinement pressure, *f*_lj_. According to the current state-of-the-art, *ε*_ju_ is defined as the actual hoop rupture strain measured in the FRP jacket, as, in most cases, it is considerably smaller than the ultimate tensile strain found from flat coupon tensile tests *ε*_FRP_. Therefore, Lam and Teng [6] established an FRP efficiency factor *k*_ε_, defined by

(4) $$\varepsilon_{\mathrm{ju}}{\;=\;\varepsilon }_{\mathrm{FRP}}\;\cdot {\;k}_{\varepsilon }.$$

Although most approaches are derived by the same basic functions, the design models show significant differences. Table 1 provides an overview of the selected, renowned models for the design of confined concrete.

**Figure 2:**
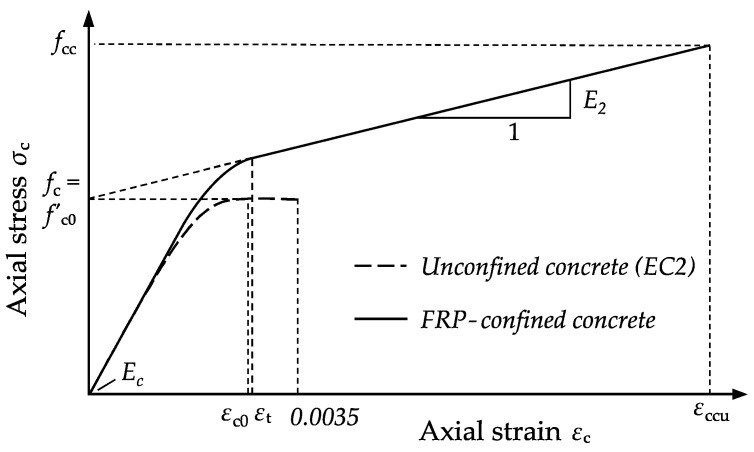
Stress–strain model for FRP-confined concrete according to Lam and Teng [6].

Most design models are used to determine the ultimate stress and strain conditions of a column under concentric compression or with comparatively small eccentricities. However, proper confinement can also provide significant strength enhancement for members subjected to combined compression and flexure. For the design of eccentrically loaded, FRP-confined columns, proper material models are essential. In general, these models use stress (σ_c_)–strain (*ε*_c_) curves with a parabolic first portion and a straight line second portion (second modulus). An example is given by the stress–strain model of Lam and Teng [6]:

(5) $$\sigma_{c}\;=\;\{\begin{array}{c} E_{c}\;\cdot {\;\varepsilon }_{c0}\;-\;\frac{{\left(E_{c}{\;-\;E}_{2}\right)}^{2}}{4\;\cdot {\;f}_{c0}}\;\cdot {\;\varepsilon }_{c0}{}^{2}\\ f_{c0}{+\;E}_{2}\;\cdot {\;\varepsilon }_{c0} \end{array}\begin{array}{c} \mathrm{if}\;0\;\leq {\;\varepsilon }_{c0}\;\leq {\;\varepsilon }_{t}\\ {\mathrm{if}\;\varepsilon }_{t}\;\leq {\;\varepsilon }_{c0}\;\leq {\;\varepsilon }_{\mathrm{ccu}} \end{array},$$

where *E*_2_ is the second modulus, *E*_c_ is the modulus of elasticity, and *ε*_t_ is the strain value at the transition between the parabolic curve and the straight-line second portion. A graphical representation of Lam and Teng’s stress–strain model is given in Figure 2.

**Figure 3:**
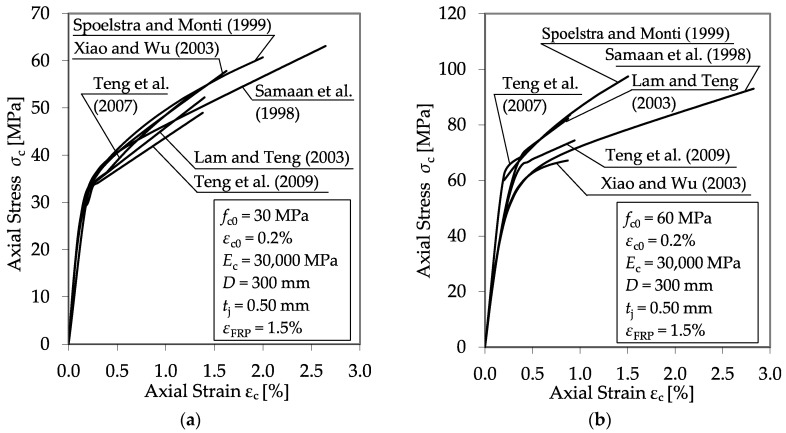
Theoretical material behavior of carbon FRP (CFRP)-confined normal strength (**a**) and high strength (**b**) concrete columns according to different models and proposals collected from the literature [6,11,13,19,32,34].

The empirical approaches for the development of design-oriented models (Table 1) mostly follow the concept of Richart et al. [31], introducing empirical confinement effectiveness coefficients *k*_1_ (ultimate stress) and *k*_2_–*k*_4_ (ultimate strain). In the majority of cases, *k*_1_ and *k*_2_–*k*_4_ are defined as constant values or are solely dependent on the maximum confining pressure *f*_lj_. These concepts lead to considerable discrepancies regarding the prediction of confined columns with different initial concrete strengths, *f*_c0_. Figure 3 shows a graphical comparison of stress–strain curves, predicted by the models listed in Table 1, for two specimens—one with a normal (30 MPa) and one with a high (60 MPa) unconfined concrete strength. Particularly for a high initial concrete strength, remarkable differences between the calculated stress–strain curves and the ultimate condition values of *f*_cc_ and *ε*_ccu_ can be seen. The discrepancies between the predicted results tend to increase significantly alongside the unconfined concrete strength.

**Figure 4:**
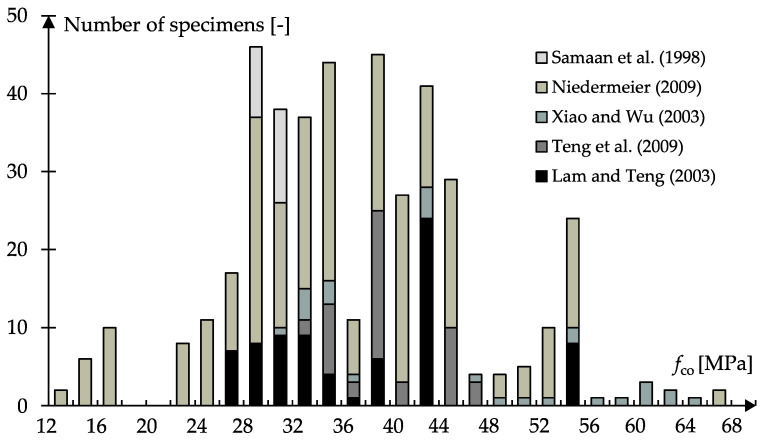
Number of specimens as a function of the initial concrete strength, *f*_c0_, used for the derivation of empirical design models for FRP-confined concrete by the authors of [6,11,13,32,33].

The relatively good correlations of the exemplary calculations with *f*_c0_ = 30 MPa may be due to the fact that most empirical design models use experimental investigations on normal-strength concrete for the derivation of the confinement effectiveness, *k*_1_ and *k*_2_–*k*_4_ (Figure 4).

Furthermore, the presented models and equations only concern the confinement effect of the CFRP jacket. The contribution of the internal transverse steel reinforcement and other effects, such as the buckling of the longitudinal steel reinforcement, are not taken into account. Only a few confinement models, e.g., Hu et al. [5], Eid and Paultre [3], Rousakis and Karabinis [35], Pellegrino and Modena [8], Teng et al. [12], or Niedermeier [33], consider the interaction between the internal lateral steel reinforcement and the external FRP jacket. The most common proposals are shown in Table 2. These models are mostly based on the basic function of Richart et al. [31] where the increase in strength and strain is not dependent on the unconfined concrete strength, *f*_c0_. 

Despite the extensive research efforts carried out in the field of FRP confinement of RC columns, there is still a substantial need for research. Particularly research regarding the determination of the confinement effectiveness coefficients as well as the interaction between the FRP-confining jacket and the internal steel reinforcement, which has thus far been considered contradictory by different design models. Furthermore, the literature lacks experimental investigations of FRP-confined RC specimens with adequate variation in different material parameters and sufficient documentation.

## 2. Experimental Investigations

### 2.1. Experimental Program

The main objective of this research program was to resolve the pending issues and knowledge gaps regarding the modeling of FRP-confined concrete revealed during the literature review. Primarily, the interaction between the FRP jacket and the transverse steel reinforcement formed part of the investigations. As described in Section 1, the existing design-oriented approaches for dual FRP–steel confinement (see, e.g., in [3,7,8,36]) show significant discrepancies. Furthermore, most experimental programs lack adequate variation in the material properties used. 

Therefore, a test program of CFRP-confined plain and RC cylinders, including the following variation parameters, was conceived:

- Diameter of the concrete cylinders
- Concrete mixture/mechanical properties of the core concrete
- Shape of the transverse steel reinforcement (i.e., tie/spiral)
- Diameter and volumetric ratio of the transverse steel reinforcement
- Mechanical properties of the transverse steel reinforcement
- Surface texture of the transverse steel reinforcement
- Volumetric ratio of the longitudinal steel reinforcement
- CFRP material
- Volumetric ratio of the CFRP jacket

In total, the program included 63 CFRP-confined plain concrete specimens and 60 CFRP-confined RC specimens with circular cross sections.

### 2.2. Materials

The following materials were used for the production of the test specimens.

#### 2.2.1. Concrete

The concrete specimens were produced using different concrete mixtures. Each series was made of concrete from the same batch. All series used CEM II 32.5 cement according to EN 197-1:2011 [37], natural aggregates with a maximum grain size of 16 mm and fly ash. The concrete mixtures were mainly designed to meet the requirements of a standard concrete with a compressive strength *f*_c0_ between 25 and 40 MPa. The properties of the hardened concrete were determined on cylinders with a diameter of 150 mm according to EN 12390-3:2009 [38].

#### 2.2.2. Steel Reinforcement

Table 3 shows the experimentally determined properties of the applied internal steel reinforcement. In most cases, steel reinforcement B500 in accordance with the German standard DIN 488-1:2009-08 [39] was used (i.e., T4, T6, T8, T10, and T12).

The variation in the mechanical properties of the transverse steel reinforcement was realized using bars with differing yield strengths (i.e., T5 and T6NR) and without ribbing (i.e., T6NR).

#### 2.2.3. Carbon Fiber-Reinforced Polymer

The confining jackets consisted of unidirectional carbon fiber (CF) sheets and a two-component, thixotropic impregnating epoxy adhesive. To ensure the variation of the material properties, three different sheets from two different manufacturers were used.

**Figure 5:**
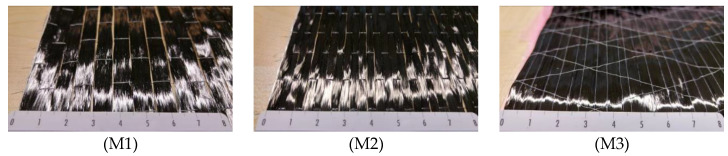
Arrangement of the fibers of the used carbon fiber (CF) sheets.

CF sheets M1 and M2 showed approximately the same material characteristics, as they originated from one manufacturer, but had a different arrangement of the carbon fibers. CF sheet M3 had a considerably higher tensile strength and rupture strain. The exact material properties, as provided by the manufacturer, are shown in Table 4, while the arrangement of the fibers of the different sheets can be seen in Figure 5. A two-component, high-strength (33.8 MPa), high-modulus (3.5 GPa) impregnating epoxy resin was used as adhesive and primer.

### 2.3. Preparation of the Test Specimens

**Figure 6:**
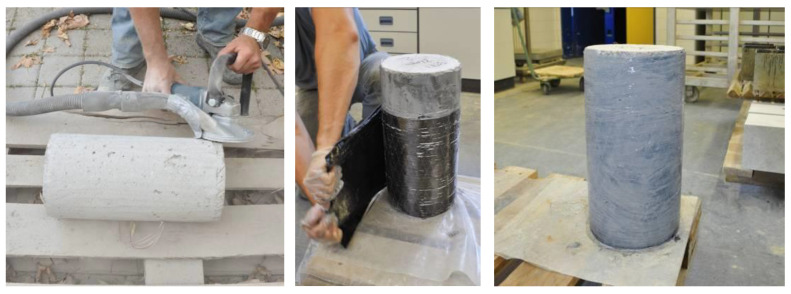
Preparation of the test specimens and application of the CFRP jacket.

Prior to the strengthening process, the concrete surface was ground until aggregates >4 mm could be seen. Additionally, the top and bottom of the cylinders were ground plane and parallel to ensure uniform load distribution. Seven days prior to the compression tests, the CFRP jacket was applied in a dry lay-up process; after the application of a primer coat to the surface of the concrete, the CF sheets were laminated continuously around the cylinders. The overlap length of the CFRPs was 100 mm, as specified by the manufacturers. The application process is shown in Figure 6.

### 2.4. Test Setup and Instrumentation

**Figure 7:**
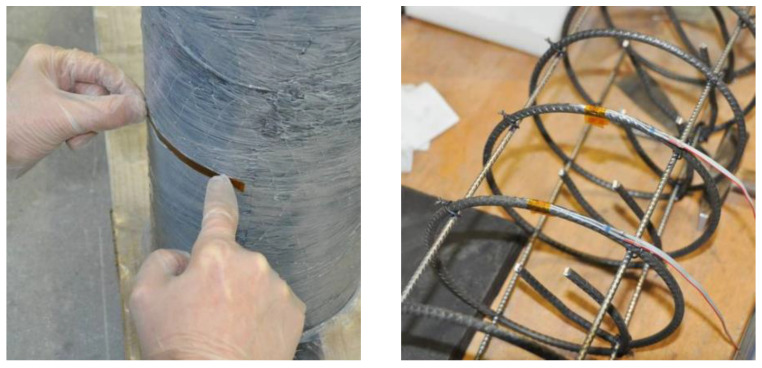
Preparation of the test specimens and application of the CFRP jacket.

The specimens were tested under uni-axial compression through monotonically applied loading using a hydraulic press with a 5000 MPa load-carrying capacity. The testing machine was set to a displacement-controlled mode with a constant rate of 0.01 mm/s. The axial displacements were measured using linear variable differential transformers (LVDTs). Lateral strains of the CFRP jacket were measured using strain gauges bonded to the specimens at mid-height. In cases where the specimens have internal reinforcement, steel strain gauges were applied on the rebar surface of the transverse reinforcement test specimen at mid-height (Figure 7).

**Figure 8:**
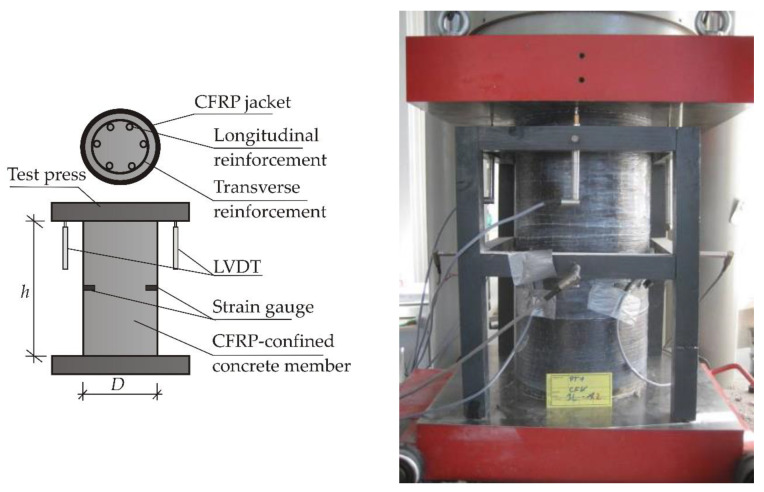
Test set-up.

Figure 8 provides a schematic description and a picture of the setup during testing.

### 2.5. Test Matrix

Table 5 shows an overview of the experimental program. The reinforced series with a diameter of 150 mm (i.e., D15-TR) were equipped with six longitudinal reinforcing bars of Type T8 according to Table 3. Series D20-TR-M2-2L-3 was split into three subseries including four (a), six (b), and eight (c) longitudinal reinforcing bars of type T12. Any further reinforced series (D20-TR, D25-SR, D25-TR, and D30-SR) were equipped with 6 longitudinal reinforcing bars of the type T12. In all reinforced series, the concrete cover was 15 mm. In series D15-P-M2-2L-2 to D-15-P-M2-2L-5, the targeted compressive strength was altered deliberately through different concrete mixtures to assess the impact of *f*_c0_ on the material behavior of the confined specimens. Furthermore, series D15-P-M2-2L-6 additionally contained a grit aggregate to examine the impact of the aggregate form and type.

## 3. Experimental Findings

### 3.1. Evaluation Methods

**Figure 9:**
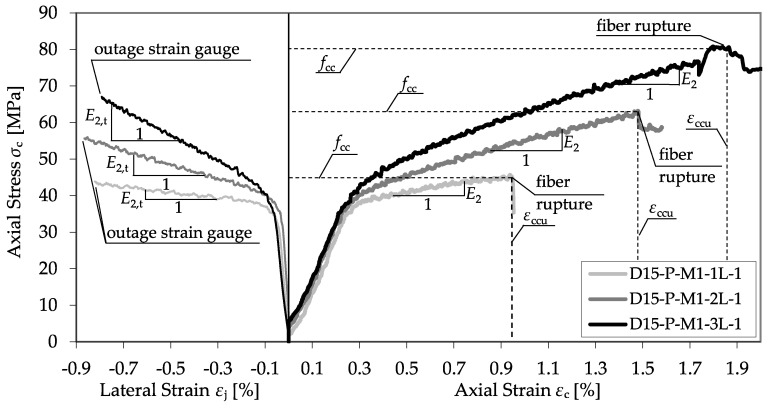
Stress–strain curves of series D15-P-M1-1L-1, D15-P-M1-2L-1, and D15-P-M1-3L-1.

The evaluation focused on the stress–strain behavior of the confined plain and RC specimens. Therefore, the axial stress was determined by the ratio of the applied load to the cross-sectional area of the concrete, disregarding the thickness of the CFPR and its possible axial resistance. Axial and lateral strains were obtained from the applied LVTDs and strain gauges. The stress–strain behavior (longitudinal and transverse) of the CFRP-confined specimens was bilinear in general, and consisted of a three-phase behavior like that predicted by the material model illustrated in Figure 2. The second modulus could be observed in the longitudinal (*E*_2_) as well as in the transverse (*E*_2,t_) direction. As an example, Figure 9 shows the stress–strain curves of single specimens of series D15-P-M1-1L-1, D15-P-M1-2L-1, and D15-P-M1-3L-1, illustrating the interrelation between *E*_2_ and the volumetric ratio of the CFRP jacket. An increase in the applied CFRP layers led to higher second moduli and higher ultimate states of strength (*f*_cc_) and strain (*ε*_ccu_).

**Figure 10:**
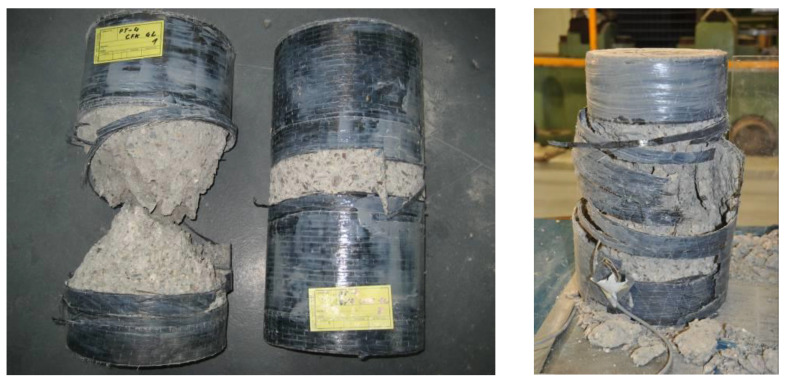
Typical failure of CFRP-confined plain concrete cylinders.

**Figure 11:**
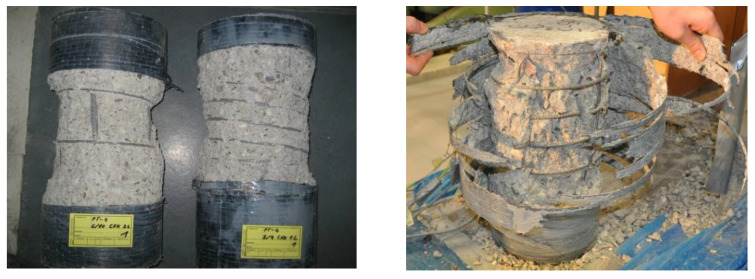
Typical failure of CFRP-confined RC cylinders.

The failure of the CFRP-confined plain or steel reinforced specimens was caused by a sudden and noisy fracture of the CFRP sheets at ultimate strength, *f*_cc_, and strain, *ε*_ccu_. Typical examples of failed confined plain and RC specimens can be seen in Figure 10 and Figure 11.

**Figure 12:**
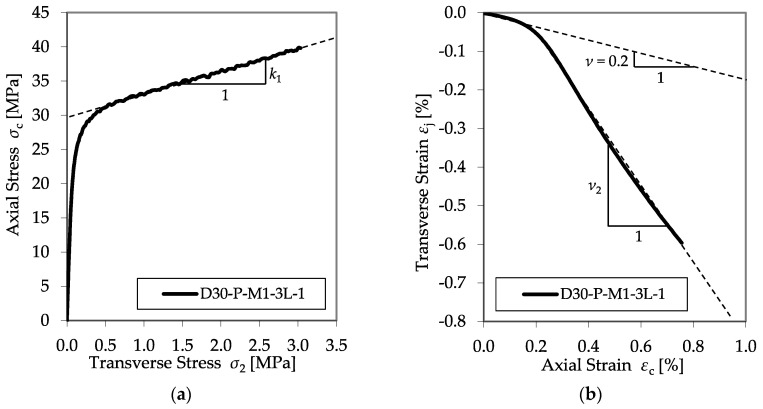
Typical axial–transverse stress (**a**) and axial–transverse strain responses (**b**).

In addition to the stress–strain relationships, the development in the comparative diagrams showing the axial–transverse strain responses and the axial–confinement stress responses of the CFRP-confined concrete specimens was an important aspect of the evaluation process. These diagrams enable the analysis of the factor *k*_1_ (cf. Equation (1)) and the second Poisson’s ratio of the confined member *ν*_2_. Typical examples are shown in Figure 12.

In most cases, the initial slopes of the axial strain and transverse strain relationships matched well the typical initial Poisson’s ratio for concrete of 0.2. As the axial strain increased, the ratio between the transverse and axial strain also increased, indicating the acceleration of the expansion of the concrete. This second linear slope describes the second Poisson’s ratio *ν*_2_. Furthermore, the axial–confinement stress response explains the design factor, *k*_1_. Once the axial stress exceeds the unconfined concrete strength, the curves converge to flatter linear relationships compared to that of the initial behavior, expressing the empirical confinement effectiveness coefficient *k*_1_.

### 3.2. CFRP-Confined Concrete Specimens

Table 6 shows the results obtained from the CFRP-confined plain concrete specimens without internal reinforcement. 

For the following analysis, the specific values *ρ*_j_, *E*_jl_, and *f*_lj_ had to be determined for each series. Set in relation to the unconfined concrete strength, the ratios *E*_jl_/*f*_c0_, *E*_jl_/*f*_c0_^2^, and *f*_lj_/*f*_c0_ can be defined (Table 7). 

**Figure 13:**
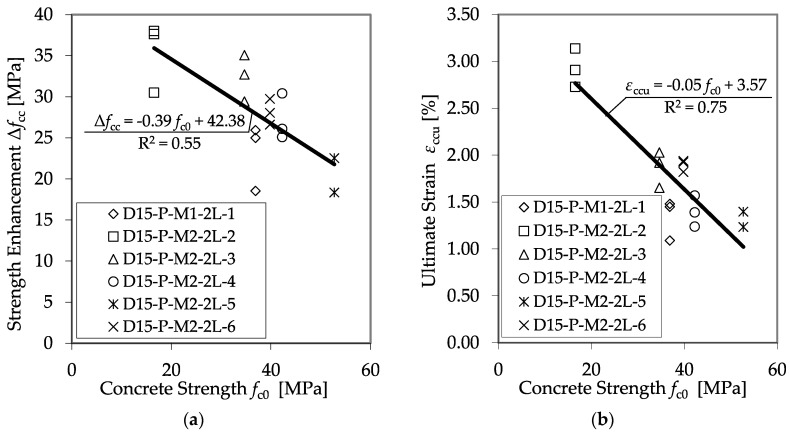
Dependence of Δ*f*_cc_ (**a**) and *ε*_ccu_ (**b**) on the unconfined concrete strength, *f*_c0_.

The variation in the diameter of the cylinder, as well as the thickness of the CFRP, led to varying volumetric ratios of the CFRP jackets, *ρ*_j_. The volumetric ratio and the material properties of the CFRP jacket define its maximum confinement pressure, *f*_lj_, as shown in Equation (3). As expected, *f*_lj_ had a significant impact on *f*_cc_ and *ε*_ccu_. Furthermore, the investigations indicated that the unconfined concrete strength, *f*_c0_, is a second impact factor. Figure 13 illustrates the dependence of the strength enhancement, Δ*f*_cc_ (Δ*f*_cc_ = *f*_cc_ − *f*_c0_) and the ultimate strain, *ε*_ccu_, on the initial concrete strength, *f*_c0_.

**Figure 14:**
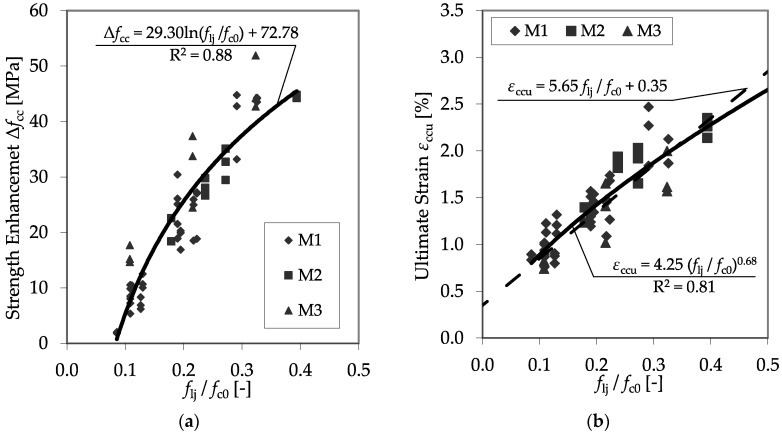
Strength enhancement Δ*f*_cc_ (**a**) and ultimate strain *ε*_ccu_ (**b**) as functions of the relationship between confinement pressure and unconfined concrete strength.

For this comparison, only *f*_c0_ was changed. Only test specimens with equal diameters (150 mm) and properties of the applied CFRP system were used, while the concrete strength, *f*_c0_, varied. An impact of *f*_c0_ on *f*_cc_ and *ε*_ccu_ can be recognized, but a sufficient correlation is pending. Therefore, the proposal of Xiao and Wu [13] was applied to involve the unconfined strength into the analysis. If *f*_l_ is set in relation to *f*_c0_, satisfying regressions for the prediction of *f*_cc_ and *ε*_ccu_ can be found. Figure 14 shows the results of all plain test specimens defined using the CFRP system, as listed in Table 6, and the regression curves for the strength enhancement, Δ*f*_cc_, and the ultimate strain, *ε*_ccu_.

The high coefficients of determination of the regression curves indicate the reliability of the ratio between confinement pressure and unconfined concrete strength to predict the load-bearing capacity of a CFRP-confined concrete member. 

**Figure 15:**
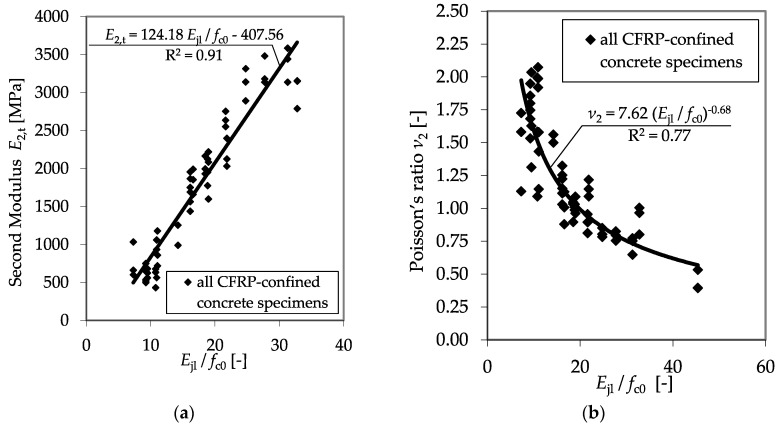
Second modulus *E*_2,t_ (**a**) and second Poisson’s ratio *ν*_2_ (**b**) as functions of the relationship between the confinement modulus and the unconfined concrete strength.

Further analysis confirmed that relating the confinement modulus *E*_jl_ to the divisor *f*_c0_ enables the prediction of *E*_2,t_, as well as *ν*_2_^,^. Figure 15 shows the results of all plain test specimens as listed in Table 6, as well as the regression curves for the second modulus *E*_2,t_ and the second Poisson’s ratio, *ν*_2_.

The comparison of the variation in the cross-sectional diameter showed no significant size effect on the FRP-confined concrete. The use of the confinement modulus *E*_jl_ and the calculated confinement pressure *f*_lj_ are sufficient for the consideration of the varying diameter.

### 3.3. FRP Rupture Strain and Accompanied Partial Safety Factors

Regarding the CFRP’s rupture strain reached by the CFRP jacket, the investigations correspond with the findings of Lam and Teng [6,23]. In almost all cases, the rupture strain was considerably lower than the ultimate tensile strain found from flat coupon tensile tests. Therefore, a factor *k*_ε_ < 1.0 should be mandatory. An overview of different approaches to determine *k*_ε_ is given in Table 8.

**Figure 16:**
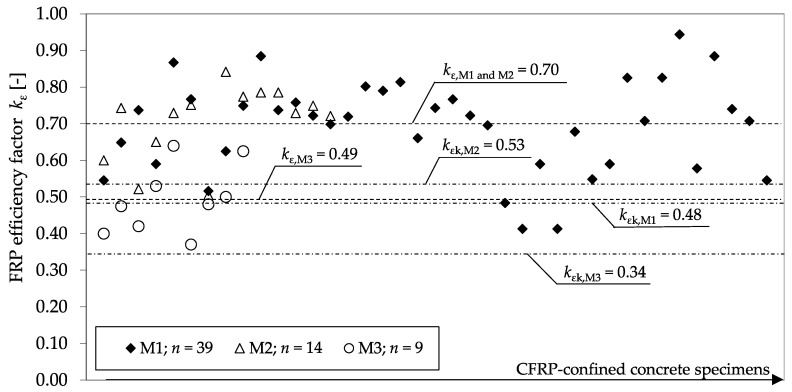
Values for *k*_ε_ determined from tests with different CFRP materials and calculated characteristic values *k*_εk_ (according to EN 1990:2002 [42]).

While most approaches suggest a common, universally valid reduction factor for CFRP systems, the conducted experimental program shows significant differences, even between the used carbon fibers. The average value for the three different CFRP systems differed remarkably between *k*_ε_ = 0.49 and *k*_ε_ = 0.70. The use of a mean value *k*_ε_, as mainly suggested in literature, can, therefore, be uncertain. Due to the large scattering of the test results, the conservative approach introduced by Niedermeier [33,40] was adopted, using characteristic values, *k*_εk_. In accordance with EN 1990:2002 [42], characteristic values for the tested specimens were determined; the results can be seen in Figure 16. In summary, the evaluation revealed the dependence of the efficiency factors *k*_ε_ on the used CFRP material.

Furthermore, the findings enabled the derivation of particular partial factors *γ*_j_ for the used CFRP materials. The approach introduced in the fib bulletin 80 [43] was used for the calculation:

(6) $$\gamma_{j}\;=\;\frac{\exp(-1.645\;\cdot {\;V}_{x})}{{\exp(-\alpha }_{R}\;\cdot \;\beta \;\cdot {\;V}_{x})}\;\cdot {\;\gamma }_{\mathrm{Rd}1}\;\cdot {\;\gamma }_{\mathrm{Rd}2}\;,$$

where *α*_R_ is the sensitivity factor (*α*_R_ = 0.8), *V*_x_ is the presumed coefficient of variation of the rupture strain *ε*_FRP_, *β* is the reliability factor (*β* = 3.8), *γ*_Rd1_ is a factor considering model uncertainties, and *γ*_Rd2_ is a factor considering geometrical uncertainties.

As shown in Table 9, the variation coefficients *V*_x_ vary remarkably between the used CFRP materials. Hence, *γ*_j_ should be determined separately for each FRP system—for instance, within a technical approval procedure.

For the derivation of the displayed partial factors according to Equation (7), *γ*_Rd1_ was predicted with a value of 1.20 because model uncertainties are comparable to that of models for shear design. In contrast, *γ*_Rd2_ was determined with a value of 1.0. For columns with a circular cross section, the geometrical uncertainties are negligible, as *k*_ε_ persisted at a constant value independent of the column diameter. 

In comparison, the calculated safety factors are significantly higher than those suggested by current recommendations, codes, and guidelines, as listed in Table 10. These partial safety factors originated from flat coupon tests of CFRP laminates and were not conditional on the application. However, this is a potential unsafe approach, as *γ*_j_ depends on *V*_x_ of the FRP jacket’s hoop strain applied to the column perimeter. The same applies for the characteristic values of the FRP strength and rupture strain.

### 3.4. CFRP-Confined Reinforced Concrete Specimens

Table 11 shows the results obtained from the tests using the CFRP-confined concrete specimens with internal reinforcement, confirming a joint confinement effect by the external CFRP confinement and internal transverse reinforcement. Dual confinement strongly increases the load-bearing capacity in general. Therefore, the confinement pressures of the CFRP jacket and the transverse steel reinforcement have to be summed according to the work in [3]:

(7) $$f_{l(j+w)}{\;=\;f}_{\mathrm{lj}}{\;+\;f}_{l,\mathrm{wy}}\;=\;\frac{1}{2}\;\cdot {\;\rho }_{j}\;\cdot {\;E}_{j}\;\cdot {\;\varepsilon }_{\mathrm{ju}}\;+\;\frac{1}{2}\;\cdot {\;\rho }_{\mathrm{st}}\;\cdot {\;f}_{y}\;\cdot {\;k}_{e}{\;\mathrm{with}\;k}_{e}\;=\;{\left(\frac{D_{c}-\;s/2}{D}\right)}^{2}{\;\mathrm{and}\;\rho }_{\mathrm{st}}=\;\frac{\pi \;\cdot \;\emptyset_{w}{}^{2}}{D_{c}\;\cdot \;s}\;,$$

where *ρ*_st_ is the transverse steel volumetric ratio, *f*_y_ is the yield stress, *k*_e_ is the coefficient of lateral and vertical efficiency of the transverse steel reinforcement according to Niedermeier [33], *D*_c_ is the horizontal center distance of the spiral or tie reinforcement, *Ø*_w_ is the diameter of the transverse steel reinforcement, and *s* is the vertical spacing between the spiral or tie bars.

For the following analysis, the provided confinement pressure and confinement stiffness had to be determined for each series. The specific values are shown in Table 12. Additionally, the cross-sectional area of the longitudinal reinforcement *A*_sl_ and the maximum stress carried by the longitudinal reinforcement during the compression test *σ*_sl_ are specified. The strength enhancement Δ*f*_cc_ is defined as Δ*f*_cc_ = *f*_cc_ − *f*_c0_ − *σ*_sl_.

**Figure 17:**
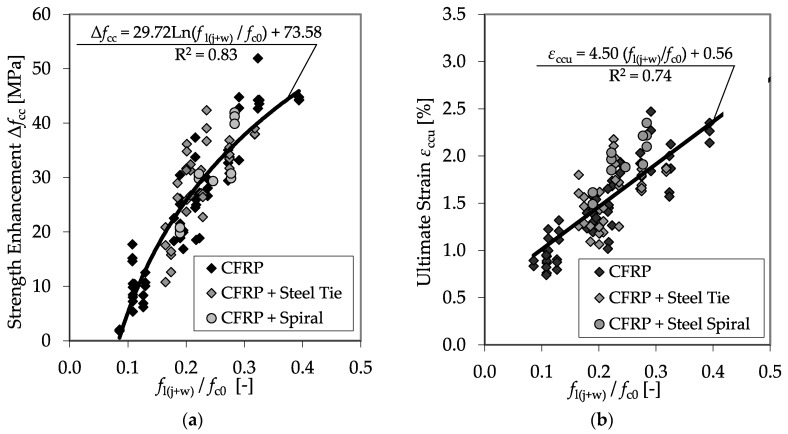
Strength enhancement (**a**), Δ*f*_cc_, and ultimate strain (**b**), *ε*_ccu_, as functions of the ratio between *f*_l(j+w)_ and *f*_c0_

In the diagrams of Figure 17, the experimental results for the strength enhancement, as well as the ultimate strain reached for both the confined plain and the RC cylinders are shown as functions of the ratio between *f*_l(j+w)_ and *f*_c0_. As for the results of the sole confined plain concrete specimens, satisfying regressions for the prediction of *f*_cc_ and *ε*_ccu_ can be found.

As observed for the plain concrete, the bearing behavior of the confined RC is defined by a decrease in the specimens’ axial rigidity. However, the transition zone is smoother and prolonged.

**Figure 18:**
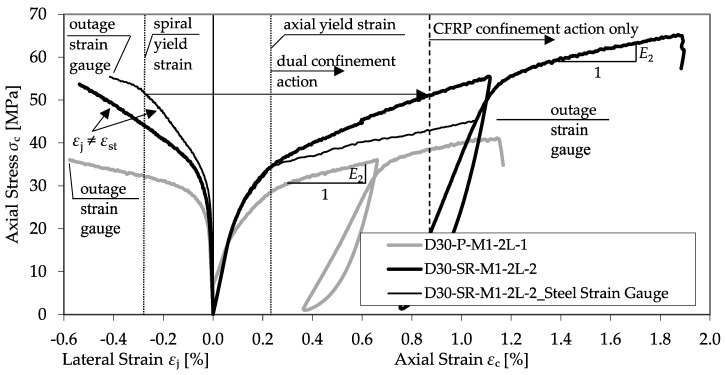
Comparison between a confined concrete specimen (D30-P-M1-2L-1) and an RC specimen (D30-SR-M1-2L-2).

Figure 18 shows the differences in bearing behavior, comparing a CFRP-confined plain concrete specimen and a column dually confined by a transverse spiral reinforcement and a CFRP jacket. In detail, a single specimen of series D30-SR-M1-2L-2 with a diameter of 300 mm and a spiral (*Ø* = 10 mm, *s* = 55 mm) was compared to a specimen of the same diameter and confinement but without reinforcement (series D30-P-M1-2L-1). As explained by Equation (7), a constant confining pressure of the yielding steel transverse reinforcement can be assumed. The second modulus is similar to *E*_2_ observed in confined plain concrete, as further strength enhancement depends on the linear elastic CFRP jacket.

**Figure 19:**
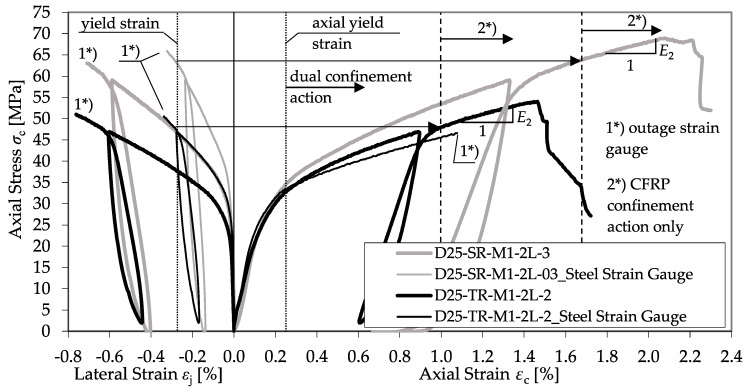
Comparison between a confined spiral-reinforced specimen (D25-SR-M1-2L-3) and a tie RC specimen (D25-TR-M1-2L-2).

In addition to the amount of transverse reinforcement, the reinforcement type was varied by the application of normal ties and heavy spirals. A comparison between both reinforcement types is given in Figure 19. Herein, a CFRP-confined specimen of series D25-SR-M1-2L-3 with a diameter of 250 mm and a spiral (*Ø* = 8 mm, *s* = 40 mm) was compared to a specimen of series D25-TR-M1-2L-2 with the same diameter and CFRP confinement but with tie reinforcement (*Ø* = 6 mm, *s* = 100 mm).

**Figure 20:**
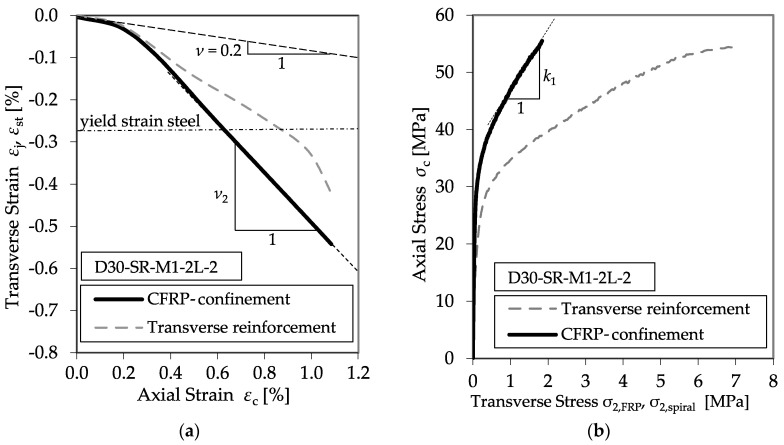
Typical axial–transverse strain (**a**) and stress (**b**) responses of external CFRP confinement and internal transverse reinforcement (specimen D30-SR-M1-2L-2)

The transition zone between the first linear increase and second linear branch, *E*_2_, of the spiral reinforced specimen is more extended. Until its yielding strength is reached, the spiral reinforcement can activate a significantly higher confinement pressure, leading to a higher *f*_cc_ and *ε*_ccu_. However, the *E*_2_ reached is almost similar. In addition, Figure 18 and Figure 19 reveal a discrepancy between the strain development of the CFRP jacket and the transverse reinforcement. Exceeding the elastic range of the concrete, the strain of the transverse reinforcement *ε*_st_ increased more slowly compared to the CFRP jacket, *ε*_j_. This behavior is contradictory to the assumptions of most material models, e.g., Hu et al. [5] or Eid and Paultre [3]. These models suppose an equal strain distribution of *ε*_j_ and *ε*_st_. Figure 20 shows the deviations in the axial–transverse strain responses and the axial–confinement stress responses for series D30-SR-M1-2L-2.

### 3.5. Impact of the Longitudinal Reinforcement on the CFRP Jacket’s Rupture Strain

Previous investigations on the impact of longitudinal reinforcement on the CFRP jacket’s rupture strain, e.g., by Pellegrino and Modena [8] and Bai et al. [45], suppose additional effects of the buckling steel bars on the reduction factor *k*_ε_. Niedermeier [33,40] followed this proposal and suggested a mean value *k*_ε_ = 0.50 and a characteristic value *k*_εk_ = 0.25. This procedure was adopted by the German Guideline for FRP Strengthening of Concrete Structures by DAfStb [30].

**Figure 21:**
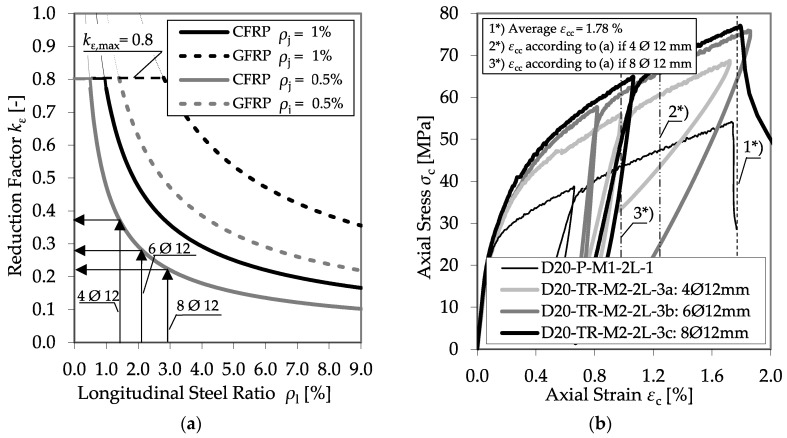
Proposal of Pellegrino and Modena [8] concerning *k*_ε_ (**a**) and a comparison between confined RC specimens with different numbers of longitudinal bars (**b**).

The experimental investigations did not confirm the assumption suggested in [8]. In general, the longitudinal reinforcement had no impact on the ultimate rupture strain of the CFRP jacket. Figure 21 shows a comparison of series D20-TR-M2-2L-3a, D20-TR-M2-2L-3b, and D20-TR-M2-2L-3c. Therein, CFRP-confined specimens with a diameter of 200 mm and the same tie configuration (*Ø* = 6 mm, *s* = 100 mm) with a different number of longitudinal reinforcing bars (*Ø* = 12 mm) were compared, showing that the number of bars differed between 4, 6, and 8. In all cases, approximately the same maximum axial strain, *ε*_ccu_, was reached. A strong impact of the longitudinal reinforcement on *ε*_ju_ should influence the confinement pressure, *f*_l_; because of this, the diagram on the left of Figure 21 explains the determination of *k*_ε_ for the three longitudinal bar configurations by using the proposal of Pellegrino and Modena [8]. As the number of bars increases, *k*_ε_ should decrease and, therefore, reduce *ε*_ccu_; however, the tests could not confirm these assumptions.

In conclusion, the reduction factor *k*_ε_ remains constant independent of the applied longitudinal reinforcement. Low reduction values such as *k*_εk_ = 0.25 are highly conservative and may provoke an unnecessary loss of load-bearing capacity.

## 4. Implementation of the Experimental Results from the Literature

### 4.1. Included Experimental Programs

The obtained test database was enlarged with the test results of Eid et al. [4], Xiao and Wu [13], Lee et al. [46], Matthys et al. [47], Lam and Teng [48,49] and Ilki et al. [50]. The sufficient documentation, including all geometrical and mechanical parameters needed for analysis, was the main reason for the specific selection. Furthermore, the listed experimental programs provide an adequate variation in initial concrete strengths and properties of the used CFRP composites. In addition, the investigations contained several CFRP-confined RC specimens and large-scaled tests. Table 13 specifies the general properties of the used materials for those experiments.

The implemented databases enabled the consideration of different FRP materials (particularly different *E*_j_), concrete mixtures with variable unconfined concrete strengths (until a high-performance area >100 MPa), and different reinforcement approaches. In Table 14 and Table 15, the collected test data regarding CFRP-confined plain and reinforced concrete specimens were collated.

In addition, Table 16 shows the collected data concerning *ν*_2_ and *k*_1_ from Xiao and Wu [13].

### 4.2. CFRP-Confined Plain Concrete Specimens

**Figure 22:**
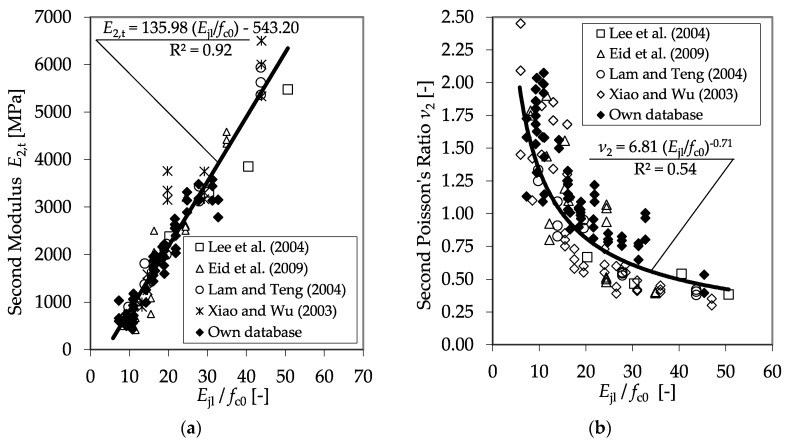
*E*_2,t_ (**a**) and *ν*_2_ (**b**) as functions of the ratio between the confinement modulus and the unconfined concrete strength including the databases in [4,13,46,48].

**Figure 23:**
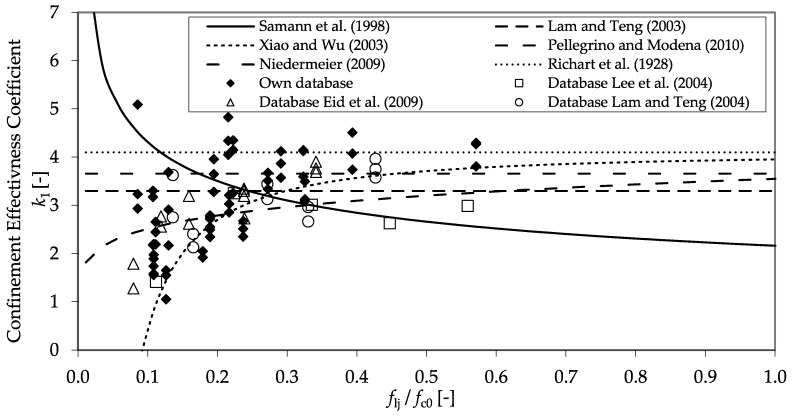
Relationship between factor *k*_1_ and the ratio between the confinement pressure and the unconfined concrete strength. Comparison of design models in [6,8,13,31,32,33] with experimental databases including those in [4,46,48].

With the collected data, the database could be significantly extended. In Figure 22, the factors *E*_2,t_ and *ν*_2,_ which are crucial for the description of the stress–strain behavior, are shown as functions of the ratio between the confinement modulus and the unconfined concrete strength. In both cases, the collected data validate the findings described in Section 3.2. Furthermore, the higher diversity of the results allowed for the assessment of a constant design factor, *k*_1_, to predict *f*_cc_. In Figure 23, all of the gathered results concerning *k*_1_ are presented as a function of the ratio *f*_l_/*f*_c0_.

**Figure 24:**
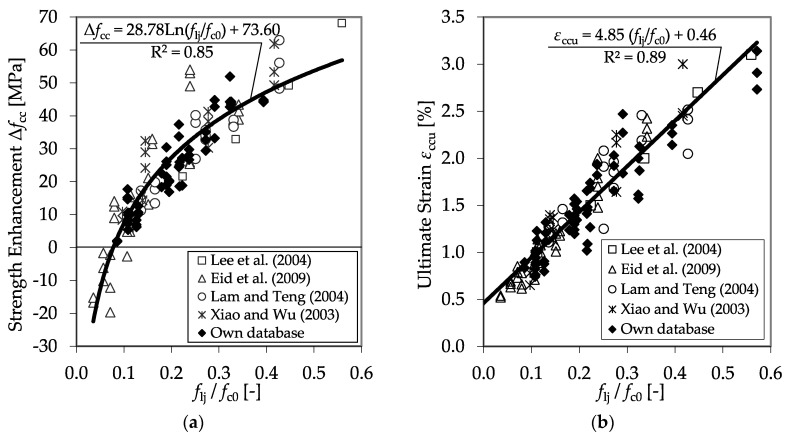
*f*_cc_ (**a**) and *ε*_ccu_ (**b**) as functions of the ratio between the confinement pressure and the unconfined concrete strength including the databases in [4,13,46,48].

Obviously, no established approach for the prediction of *k*_1_ can fit the test database, exhibiting a considerable scatter. In conclusion, the design factor *k*_1_ has to be reflected critically in general. The gathered data indicates an advantage in using the ratio between the confinement pressure and unconfined concrete strength to predict *f*_cc_ and *ε*_ccu_, as seen in Figure 24. 

### 4.3. CFRP-Confined Reinforced Concrete Specimens

**Figure 25:**
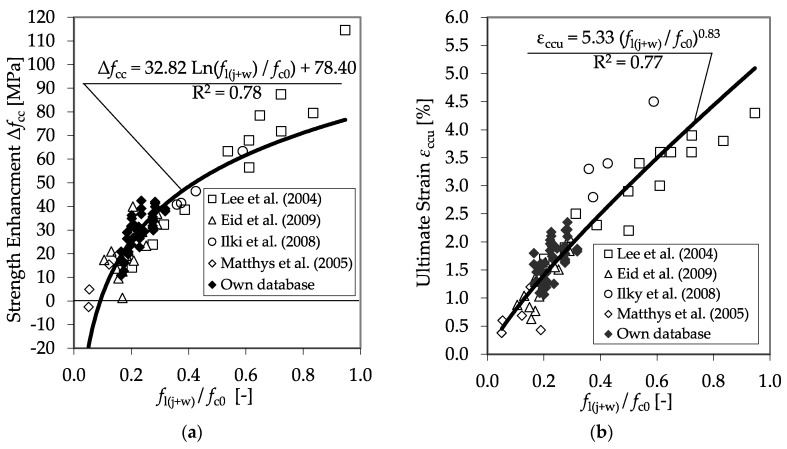
Strength enhancement Δ*f*_cc_ (**a**) and maximum strain *ε*_ccu_ (**b**) as functions of the ratio between *f*_l(j+w)_ and *f*_c0_ including the databases of [4,46,47,50].

Only few references regarding tests with CFRP confined RC specimens offer sufficient and comprehensive data concerning the applied CFRP system, the arrangement and construction of the longitudinal and transverse reinforcement as well as detailed information on the reached *f*_cc_ and *ε*_ccu_. However, the considered data sets regarding CFRP confined RC columns only included 39 test results. Nevertheless, combined with the experimental results described in Section 3.4, the gathered database enabled satisfying regressions for the prediction of *f*_cc_ and *ε*_ccu_. Figure 25 shows the determined dependency of Δ*f*_cc_ and *ε*_ccu_ on the ratio between the total confinement pressure *f*_l(j+w)_ and the unconfined concrete strength *f*_c0_.

The extent of the tested ratios *f*_l(j+w)_/*f*_c0_ covered by the experimental results could be enlarged to values close to *f*_l(j+w)_/*f*_c0_ = 1.0. In this case, the confinement pressure exceeded the unconfined concrete strength. The correlations in Figure 25 show the applicability of the ratio between the confinement pressure and the unconfined concrete strength for the description of the behavior of the CFRP-confined RC material.

## 5. Model for CFRP-Confined Plain and Reinforced Concrete

### 5.1. Ultimate Concrete Strength and Accompanied Axial Strain

**Figure 26:**
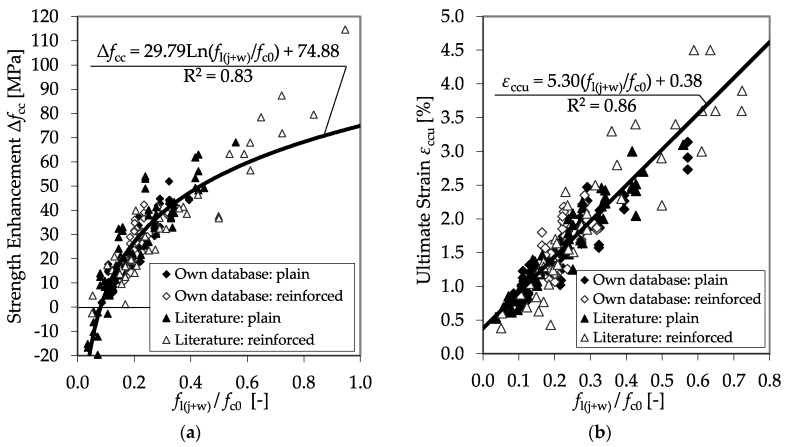
Strength enhancement (**a**), Δ*f*_cc_, and maximum strain (**b**), *ε*_ccu_, as functions of the ratio between *f*_l(j+w)_ and *f*_c0_ including the databases in [4,13,46,47,48,49,50] (cf. Table 14 and Table 15).

For an overall evaluation of the achievable ultimate concrete strength, *f*_cc_, and strain, *ε*_ccu_, the results of the CFRP-confined plain concrete specimens, as well as the CFRP-confined RC specimens, were considered in a unified regression analysis. The database and the regression results are presented in Figure 26. In conclusion, general equations for the prediction of *f*_cc_ and *ε*_ccu_ could be determined as the following,

(8) $$f_{\mathrm{cc}}{\;=\;f}_{c0}\;+\;30\;\cdot \;\ln\;\left(\frac{f_{l(j+w)}}{f_{c0}}\right)\;+\;75\;[\mathrm{MPa}],$$

(9) $$\varepsilon_{\mathrm{ccu}}{\;=\;\varepsilon }_{c0}\;\cdot \;1.75\;+\;0.05\;\cdot \;\frac{f_{l(j+w)}}{f_{c0}}\;[\%].$$

To allow the implementation of the results in modern limit state design concepts, Equation (10) presents an approach for the calculation of the characteristic strength, *f*_cck_:

(10) $$f_{\mathrm{cck}}{\;=\;f}_{\mathrm{ck}}\;+\;30\;\cdot \;\ln\;\left(\frac{f_{\mathrm{lk}(j+w)}}{f_{c0}}\right)\;+\;63\;\mathrm{if}\;0.75\;\geq \;\frac{f_{\mathrm{lk}(j+w)}}{f_{c0}}\;\geq \;{0.125\;\mathrm{with}\;f}_{\mathrm{lk}(j+w)}{=\;E}_{\mathrm{jl}}\;\cdot {\;\varepsilon }_{\mathrm{juk}}\;+\;\frac{1}{2}\;\cdot {\;\rho }_{\mathrm{st}}\;\cdot {\;f}_{\mathrm{yk}}\;\cdot {\;k}_{e}\;[\mathrm{MPa}].$$

where *f*_ck_ is the characteristic concrete compressive strength, *ε*_juk_ is the characteristic rupture strain of the FRP jacket in the application of confinement (*ε*_juk_ = *ε*_FRP_ · *k*_εk_), and *f*_yk_ is the characteristic yield stress of the steel reinforcement. 

The limitations ensure that the calculation is within boundaries of the gathered experimental results. 

### 5.2. Stress–Strain Relationships

For the design of a stress–strain model, the stress–strain relationships proposed by Lam and Teng [6] (Equation (5)) were adopted. Analysis of the experimental results revealed a significant dependency between the second modulus in the transverse direction, *E*_2,t_, the second Poison’s ratio, *ν*_2_, and the second modulus in the axial direction, *E*_2_. Therefore, the following equations for the prediction of *E*_2_ can be proposed,

(11) $$E_{2,t}\;=\;135\;\cdot \;\frac{E_{\mathrm{jl}}}{f_{c0}}\;\;\;550\;[\mathrm{MPa}],$$

(12) $$v_{2}\;=\;7\;\cdot \;{\left(\frac{E_{\mathrm{jl}}}{f_{c0}}\right)}^{-0.7},$$

(13) $$E_{2}{\;=\;E}_{2,t}\;\cdot {\;v}_{2}.$$

Furthermore, the transition point between the parabolic curve and the straight-line second portion, *ε*_t_, can be described by the following equations,

(14) $$f_{c}^{\;*}{\;=\;f}_{\mathrm{cc}}{-\;E}_{2}\;\cdot {\;\varepsilon }_{\mathrm{ccu}}\;,$$

(15) $$\varepsilon_{t}\;=\;\frac{2\;\cdot {\;f}_{c}^{\;*}}{E_{c}\;{-\;E}_{2}}.$$

Finally, the stress–strain relationship is given as follows,

(16) $$\sigma_{c}\;=\;\{\begin{array}{c} E_{c}\;\cdot {\;\varepsilon }_{c}\;-\;\frac{{\left(E_{c}{\;-\;E}_{2}\right)}^{2}}{4\;\cdot {\;f}_{c}^{*}}\;\cdot {\;\varepsilon }_{c}{}^{2}\\ f_{c}^{*}{+\;E}_{2}\;\cdot {\;\varepsilon }_{c} \end{array}\begin{array}{c} \mathrm{if}\;0\;\leq {\;\varepsilon }_{c}\;\leq {\;\varepsilon }_{t}\\ {\mathrm{if}\;\varepsilon }_{t}\;\leq {\;\varepsilon }_{c}\;\leq {\;\varepsilon }_{\mathrm{ccu}} \end{array},$$

## 6. Conclusions

FRP materials are gaining importance in construction. Especially for strengthening purposes, fiber-reinforced polymers show great potential [51,52]. FRP confinement can significantly increase the strength and ductility of concrete and RC. The present study confirmed the bilinear stress–strain model proposed by Lam and Teng [6] for confined plain and reinforced concrete. For enhancement of the ultimate strength and accompanied axial strains, the proposal of Xiao and Wu [13] using the ratio between the confinement modulus, *E*_jl_, and the unconfined concrete strength, *f*_c0_, proved to be the most correlated approach. The effect of a dual confinement on the stress–strain behavior could be explained by the individual confinement pressure provided by the CFRP jacket and the transverse steel reinforcement. Based on the model of Lam and Teng, an approach for the calculation of *f*_cc_, *ε*_ccu_, and *E*_2_ could be developed. Furthermore, the findings led to additional knowledge concerning the prediction (in accordance with the limit state method) of the CFRP’s hoop strain, *ε*_ju_, and the related partial factor, *γ*_j_. However, further research efforts are still pending. In particular, the confinement of low-strength concrete, as well as substandard concrete, was not examined in the current study. Furthermore, the effect of particularly high confinement pressures exceeding the unconfined concrete strength has yet not been sufficiently considered.

## Figures and Tables

**Table 1:** Different approaches to predict *f*_cc_ and *ε*_ccu_ of confined concrete columns.

Authors	Confined Concrete Compressive Strength *f*_cc_	Ultimate Axial Compressive Strain *ε*_ccu_
Richart et al. (1928) [31]	$f_{\mathrm{cc}}{=f}_{c0}{+k}_{1}\;\cdot {\;f}_{\mathrm{lj}}$	-
	$k_{1}=4.1$	
Samaan et al. (1998) [32]	$f_{\mathrm{cc}}{=f}_{c0}{+k}_{1}\;\cdot {\;f}_{\mathrm{lj}}$ $k_{1}=6.0\;\cdot {\;f}_{\mathrm{lj}}{}^{-0.3}$	$\varepsilon_{\mathrm{ccu}}=\frac{{\;f}_{\mathrm{cc}}{+f}_{0}}{E_{2}}$
		$E_{2}=245.61\;\cdot {\;f}_{c0}^{\;0.2}+1.3456\;\cdot \;\frac{{\;E}_{j}{+t}_{j}}{D}$
		$f_{0}=0.872\;\cdot {\;f}_{c0}+0.371\;\cdot {\;f}_{\mathrm{lj}}+6.258$
Xiao and Wu (2003) [13]	$f_{\mathrm{cc}}=\alpha \;\cdot {\;f}_{c0}{+k}_{1}\;\cdot {\;f}_{\mathrm{lj}}$	$\varepsilon_{\mathrm{ccu}}=\frac{{\;\varepsilon }_{\mathrm{ju}}}{v_{2}}=\frac{{\;\varepsilon }_{\mathrm{ju}}}{10\;\cdot \;{\left(f_{c0}/E_{\mathrm{jl}}\right)}^{0.9}}$
	$k_{1}=4.1\;-\;0.45\;\cdot {\left(\frac{f_{c0}^{\;2}}{E_{\mathrm{jl}}}\right)}^{1.4}\mathrm{with}\;\alpha \;\approx \;1.1$	
Lam and Teng (2003) [6]	$f_{\mathrm{cc}}{=f}_{c0}{+k}_{1}\;\cdot {\;f}_{\mathrm{lj}}$	$\varepsilon_{\mathrm{ccu}}{=\varepsilon }_{c0}\;\cdot \;1.75{+\varepsilon }_{c0}\;\cdot \;12\;\cdot \;\frac{{\;f}_{\mathrm{lj}}}{f_{c0}}\;\cdot {\left(\frac{\varepsilon_{\mathrm{ju}}}{\varepsilon_{c0}}\right)}^{0.45}$
	$k_{1}=3.3$	
Teng et al. (2009) [11]	$f_{\mathrm{cc}}=\{\begin{array}{c} f_{c0}{+f}_{c0}\;\cdot \;3.5\;\cdot \;\left(\rho_{k}-0.01\right)\;\cdot {\;\rho }_{\varepsilon }\\ f_{c0} \end{array}\begin{array}{c} {\mathrm{if}\;\rho }_{k}\;\geq \;0.01\\ {\mathrm{if}\;\rho }_{k}\;<\;0.01 \end{array}$	$\varepsilon_{\mathrm{ccu}}{=\varepsilon }_{c0}\;\cdot \;1.75{+\varepsilon }_{c0}\;\cdot \;6.5\;\cdot {\;\rho }_{k}{}^{0.8}\;\cdot {\;\rho }_{\varepsilon }{}^{1.45}$
	$\rho_{k}=\frac{2\;\cdot {\;E}_{j}\;\cdot {\;t}_{j}}{\left(f_{c0}/\varepsilon_{c0}\right)\;\cdot \;D}{\;\mathrm{and}\;\rho }_{\varepsilon }=\frac{\varepsilon_{\mathrm{ju}}}{\varepsilon_{c0}}$	
Niedermeier (2009) [33]	$f_{\mathrm{cc}}{=f}_{c0}{+k}_{1}\;\cdot {\;f}_{\mathrm{lj}}$	$\varepsilon_{\mathrm{ccu}}{=\varepsilon }_{c0}\;\cdot \;1.75{+\varepsilon }_{c0}\;\cdot \;19\;\cdot \;\frac{{\;f}_{\mathrm{lj}}}{f_{c0}}$
	$k_{1}=3.66$	

**Table 2:** Different approaches to predict *f*_cc_ and *ε*_ccu_ of CFRP-confined reinforced concrete (RC) columns.

Authors	Confined Concrete Compressive Strength *f*_cc_	Ultimate Axial Compressive Strain *ε*_ccu_
Eid and Paultre (2008) [3]	$f_{\mathrm{cc}}{=f}_{c0}{+k}_{1}\;\cdot \;\left(f_{\mathrm{lj}}+f_{l,\mathrm{wy}}\right)$	$\varepsilon_{\mathrm{ccu}}{=\varepsilon }_{c0}\;\cdot \;1.56{+\varepsilon }_{c0}\;\cdot \;12\;\cdot \;\left(\frac{f_{\mathrm{lj}}}{f_{c0}}+\frac{f_{l,\mathrm{wy}}}{f_{c0}}\right)\;\cdot \;{\left(\frac{\varepsilon_{\mathrm{ju}}}{\varepsilon_{c0}}\right)}^{0.45}$
	$k_{1}=3.3$	
Pellegrino and Modena (2010) [8]	$f_{\mathrm{cc}}{=f}_{c0}{+k}_{1}\;\cdot \left(f_{\mathrm{lj}}{+f}_{l,\mathrm{wy}}\;\cdot \;\frac{A_{\mathrm{cc}}}{A_{c}}\right)\;$	$\varepsilon_{\mathrm{ccu}}{=\varepsilon }_{c0}\;\cdot {\;2+\varepsilon }_{c0}\;\cdot \;B\;\cdot \;\frac{\left(f_{\mathrm{lj}}{+f}_{l,\mathrm{wy}}\;\cdot \;\frac{A_{\mathrm{cc}}}{A_{c}}\right)}{f_{c0}}$
	$k_{1}=A\;\cdot \;{\left[\frac{\left(f_{\mathrm{lj}}{+f}_{l,\mathrm{wy}}\;\cdot \;\frac{A_{\mathrm{cc}}}{A_{c}}\right)}{f_{c0}}\right]}^{-\alpha }$	
Niedermeier (2009) [33]	$f_{\mathrm{cc}}{=f}_{c0}{+k}_{1}\;\cdot \;\left[f_{\mathrm{lj}}+\left(f_{l,\mathrm{wy}}-\;\Delta p\right)\cdot {\left(\frac{D_{c}\;-\;s/2}{D}\right)}^{2}\right]$	$\varepsilon_{\mathrm{ccu}}{=\varepsilon }_{c0}\;\cdot \;1.75{+\varepsilon }_{c0}\;\cdot \;19\;\cdot \;\left(\frac{{\;f}_{\mathrm{lj}}}{f_{c0}}+\frac{f_{l,\mathrm{wy}}}{f_{c0}}\;-\;\frac{\Delta p}{f_{c0}}\right)$
	$k_{1}=3.66$	

Abbreviations: *f*_l,wy_ = confining pressure provided by transverse reinforcement; *A*_cc_ = area of core of section enclosed by the center lines of the perimeter spiral or tie; *A*_c_ = column cross section; *A*, *B*, and *α* = empirical parameters; *D*_c_ = horizontal center distance of the spiral or tie reinforcement; Δ*p* = reduction of confinement pressure between the core section and the concrete cover; *s* = vertical spacing between spiral or tie bars.

**Table 3:** Properties of the used steel reinforcement (mean values).

Type	Nominal Diameter	Ribbing	Yield Strength *f*_ym_	Tensile Strength *f*_tm_	Modulus of Elasticity	Rupture Strain
	[mm]	[-]	[MPa]	[MPa]	[GPa]	[%]
T4	4	yes	550	610	196	8
T6	6					
T8	8					
T10	10					
T12	12		500	608	194	14
T5	5		670	725	205	-
T6NR	6	no	730	760	-	12

**Table 4:** Properties of used CFRP materials.

CFRP type	Density	Axial Tensile Strength	Axial Modulus of Elasticity	Rupture Strain (axial)	Weight Per Square Meter
[-]	[g/m^3^]	[MPa]	[GPa]	[%]	[g/m^2^]
M1	1.80	3900	230	1.70	200
M2	1.80	4100	230	1.78	220
M3	1.79	4800	240	2.00	200

**Table 5:** Experimental program.

Series (3 specimens)	Concrete Strength	Dia Meter	Height	CFRP Confinement			Transverse Reinforcement		
	*f*_c0_	*D*	*h*	Material	Layers	*t*_j_	Type	*s*	Geometry
	[MPa]	[mm]	[mm]		[-]	[mm]		[mm]	
D15-P-M1-1L-1	36.9	150	300	M1	1	0.111	-	-	-
D15-P-M1-1L-2	36.9	150	300	M1	1	0.111	-	-	-
D15-P-M1-2L-1	36.9	150	300	M1	2	0.222	-	-	-
D15-P-M2-2L-2	16.5	150	300	M2	2	0.244	-	-	-
D15-P-M2-2L-3	34.7	150	300	M2	2	0.244	-	-	-
D15-P-M2-2L-4	42.3	150	300	M2	2	0.222	-	-	-
D15-P-M2-2L-5	52.7	150	300	M2	2	0.244	-	-	-
D15-P-M2-2L-6	39.8	150	300	M2	2	0.244	-	-	-
D15-P-M1-3L-1	36.9	150	300	M1	3	0.333	-	-	-
D15-TR-M1-2L-1	42.3	150	300	M1	2	0.222	T6	100	Tie
D15-TR-M1-2L-2	42.3	150	300	M1	2	0.222	T6	50	Tie
D20-P-M1-1L-1	27.0	200	400	M1	1	0.111	-	-	-
D20-P-M3-1L-2	24.5	200	400	M3	1	0.112	-	-	-
D20-P-M1-2L-1	27.0	200	400	M1	2	0.222	-	-	-
D20-P-M3-2L-2	24.5	200	400	M3	2	0.223	-	-	-
D20-P-M1-3L-1	27.0	200	400	M1	3	0.444	-	-	-
D20-P-M3-3L-2	24.5	200	400	M3	3	0.447	-	-	-
D20-TR-M1-2L-1	27.0	200	400	M1	2	0.222	T4	175	Tie
D20-TR-M1-2L-2	27.0	200	400	M1	2	0.222	T6	175	Tie
D20-TR-M2-2L-3a	28.0	200	400	M2	2	0.244	T6	100	Tie
D20-TR-M2-2L-3b	28.0	200	400	M2	2	0.244	T6	100	Tie
D20-TR-M2-2L-3c	28.0	200	400	M2	2	0.244	T6	100	Tie
D20-TR-M2-2L-4	28.0	200	400	M2	2	0.244	T6	50	Tie
D20-TR-M2-1L-1	24.5	200	400	M2	1	0.122	T6	75	Tie
D20-TR-M2-1L-2	24.5	200	400	M2	1	0.122	T6NR	75	Tie
D20-TR-M2-1L-3	24.5	200	400	M2	1	0.122	T5	50	Tie
D25-P-M1-1L-1	28.1	250	500	M1	1	0.111	-	-	-
D25-P-M1-2L-1	38.0	250	500	M1	2	0.222	-	-	-
D25-P-M1-3L-1	38.0	250	500	M1	3	0.333	-	-	-
D25-P-M1-4L-1	33.0	250	500	M1	4	0.444	-	-	-
D25-SR-M1-1L-1	33.0	250	500	M1	1	0.111	T8	40	Spiral
D25-SR-M1-2L-1	39.0	250	500	M1	2	0.222	T8	40	Spiral
D25-SR-M1-2L-2	28.1	250	500	M1	2	0.222	T10	40	Spiral
D25-SR-M1-2L-3	31.2	250	1000	M1	2	0.222	T8	40	Spiral
D25-SR-M1-3L-1	39.0	250	500	M1	3	0.333	T8	40	Spiral
D25-TR-M1-2L-1	33.0	250	500	M1	2	0.222	T6	100	Tie
D25-TR-M1-2L-2	31.2	250	1000	M1	2	0.222	T6	100	Tie
D30-P-M1-2L-1	30.8	300	600	M1	2	0.222	-	-	-
D30-P-M1-3L-1	30.8	300	600	M1	3	0.333	-	-	-
D30-SR-M1-2L-1	31.0	300	600	M1	2	0.222	T10	40	Spiral
D30-SR-M1-2L-2	31.0	300	600	M1	2	0.222	T10	55	Spiral

**Table 6:** Test results of CFRP-confined plain concrete specimens.

Series	Specimens	*f*_c0_	*f*_cc_	*ε*_ccu_	*E*_2,t_	*E*_2_	*ν*_2_	*k*_1_	*k*_ε_
		[MPa]	[MPa]	[%]	[MPa]	[MPa]	[-]	[-]	[-]
D15-P-M1-1L-1	1	36.9	42.23	0.761	497	960	1.857	1.581	0.546
	2		45.34	0.939	750	1112	1.747	2.196	0.649
	3		47.39	1.017	647	1094	1.681	1.893	0.737
	***Mean:***		**44.99**	**0.906**	**631**	**1055**	**1.762**	**1.890**	**0.644**
D15-P-M1-1L-2	1	36.9	44.90	0.868	670	1039	1.532	1.974	0.590
	2		46.72	1.001	536	999	1.800	1.553	0.867
	3		44.11	0.890	517	1008	1.948	1.739	0.767
	***Mean:***		**45.24**	**0.920**	**574**	**1015**	**1.760**	**1.755**	**0.741**
D15-P-M1-2L-1	1	36.9	55.43	1.089	1996	2079	1.033	3.031	0.516
	2		61.87	1.450	2166	2018	0.897	3.210	0.625
	3		62.82	1.480	1932	2055	1.047	2.855	0.749
	***Mean:***		**60.04**	**1.340**	**2031**	**2051**	**0.992**	**3.032**	**0.630**
D15-P-M2-2L-2	1	16.5	54.16	3.138	3273	1209	0.394	4.270	0.600
	2		54.53	2.908	2854	1292	0.533	3.807	0.743
	3		47.02	2.730	3120	1266	0.395	4.295	0.522
	***Mean:***		**51.90**	**2.925**	**3082**	**1256**	**0.441**	**4.124**	**0.622**
D15-P-M2-2L-3	1	34.7	64.07	1.652	2553	1895	0.811	3.339	0.651
	2		67.37	1.920	2754	1862	0.956	3.674	0.729
	3		69.73	2.030	2634	1757	0.894	3.514	0.752
	***Mean:***		**67.06**	**1.867**	**2647**	**1838**	**0.887**	**3.509**	**0.711**
D15-P-M2-2L-4	1	42.3	72.68	1.570	1867	2023	1.115	2.715	0.885
	2		67.36	1.240	1750	2135	1.325	2.495	0.737
	3		68.36	1.390	1956	2221	1.253	2.789	0.758
	***Mean:***		**69.47**	**1.400**	**1858**	**2126**	**1.231**	**2.666**	**0.793**
D15-P-M2-2L-5	1	52.7	75.25	1.397	1255	1470	1.499	2.046	0.785
	2		71.07	1.235	989	1291	1.561	1.919	0.785
	***Mean:***		**73.16**	**1.316**	**1122**	**1381**	**1.530**	**1.983**	**0.785**
D15-P-M2-2L-6	1	39.8	69.55	1.820	1949	1758	1.009	2.514	0.505
	2		66.42	1.938	1773	1602	1.031	2.352	0.841
	3		67.85	1.926	2124	1672	0.987	2.676	0.774
	***Mean:***		**67.94**	**1.895**	**1949**	**1677**	**1.009**	**2.514**	**0.707**
D15-P-M1-3L-1	1	36.9	81.16	1.867	3180	2672	0.825	3.125	0.722
	2		80.43	1.869	3482	2432	0.754	3.497	0.699
	3		81.05	2.125	3137	2350	0.795	3.087	0.719
	***Mean:***		**80.88**	**1.954**	**3266**	**2485**	**0.791**	**3.236**	**0.713**
D20-P-M1-1L-1	1	27.0	36.68	1.128	559	928	1.626	2.189	0.802
	2		37.39	1.226	679	893	1.311	2.654	0.790
	3		36.66	1.000	625	1066	2.035	2.446	0.814
	***Mean:***		**36.91**	**1.118**	**621**	**962**	**1.657**	**2.430**	**0.802**
D20-P-M3-1L-2	1	24.5	39.17	0.824	563	1230	2.074	2.177	0.400
	2		42.25	0.949	932	1447	1.986	3.174	0.475
	3		39.73	0.741	1059	1816	1.920	3.303	0.420
	***Mean:***		**40.38**	**0.838**	**851**	**1498**	**1.993**	**2.885**	**0.432**
D20-P-M1-2L-1	1	27.0	45.81	1.266	1600	1810	1.089	3.253	0.661
	2		54.16	1.738	2220	2057	0.963	4.349	0.743
	3		53.99	1.681	2084	2017	0.961	4.150	0.767
	***Mean:***		**51.32**	**1.562**	**1968**	**1961**	**1.004**	**3.917**	**0.724**
D20-P-M3-2L-2	1	24.5	58.29	1.411	2126	2065	1.146	4.337	0.530
	2		61.90	1.653	2032	1977	1.218	4.048	0.640
	3		48.99	1.018	2399	2597	1.091	4.825	0.370
	***Mean:***		**56.39**	**1.361**	**2186**	**2213**	**1.152**	**4.403**	**0.513**
D20-P-M1-3L-1	1	27.0	71.72	2.140	3584	2705	0.752	4.509	0.729
	2		71.15	2.264	3136	2305	0.772	3.738	0.749
	3		71.30	2.350	3440	2254	0.648	4.077	0.721
	***Mean:***		**71.39**	**2.251**	**3387**	**2421**	**0.724**	**4.108**	**0.733**
D20-P-M3-3L-2	1	24.5	67.24	1.614	3151	2244	0.802	4.128	0.480
	2		68.77	1.570	2788	2457	1.004	3.597	0.500
	3		76.45	2.000	3156	2286	0.966	4.146	0.625
	***Mean:***		**70.82**	**1.728**	**3032**	**2329**	**0.924**	**3.957**	**0.535**
D25-P-M1-1L-1	1	28.1	30.11	0.834	600	1027	1.724	2.933	0.722
	2		29.92	0.893	660	1049	1.582	3.231	0.696
	3		29.85	0.894	1033	1202	1.130	5.088	0.484
	***Mean:***		**29.96**	**0.874**	**764**	**1093**	**1.479**	**3.751**	**0.634**
D25-P-M1-2L-1	1	38.0	44.87	0.798	675	991	1.994	1.650	0.413
	2		46.33	0.905	634	1000	1.584	1.550	0.590
	3		44.20	0.877	429	550	1.091	1.050	0.413
	***Mean:***		**45.13**	**0.860**	**579**	**847**	**1.556**	**1.417**	**0.472**
D25-P-M1-3L-1	1	38.0	59.54	1.511	1564	1820	1.151	2.551	0.678
	2		56.89	1.300	1692	1727	1.030	2.759	0.548
	3		56.94	1.195	1437	1709	1.224	2.343	0.590
	***Mean:***		**57.79**	**1.335**	**1564**	**1752**	**1.135**	**2.551**	**0.605**
D25-P-M1-4L-1	1	33.0	75.80	2.270	3140	2448	0.804	3.870	0.826
	2		66.20	1.840	2890	2249	0.850	3.571	0.708
	3		77.80	2.470	3314	2503	0.783	4.121	0.826
	***Mean:***		**73.27**	**2.193**	**3115**	**2400**	**0.812**	**3.854**	**0.787**
D30-P-M1-2L-1	1	30.8	41.50	1.206	719	1152	1.580	2.167	0.944
	2		40.85	1.115	1178	1476	1.146	3.690	0.578
	3		43.33	1.319	860	1371	1.432	2.909	0.885
	***Mean:***		**41.89**	**1.213**	**919**	**1333**	**1.386**	**2.922**	**0.802**
D30-P-M1-3L-1	1	30.8	50.75	1.459	1657	1859	1.126	3.280	0.740
	2		51.08	1.539	1852	1869	1.007	3.645	0.708
	3		47.68	1.345	1991	1876	0.880	3.957	0.546
	***Mean:***		**49.84**	**1.448**	**1833**	**1868**	**1.004**	**3.627**	**0.665**

**Table 7:** Specific values for the CFRP-confined plain concrete specimens.

Series	*ρ*_j_	*f*_lj_	*E*_jl_	*E*_jl_/*f*_c0_	*E*_jl_/*f*_c0_^2^	*f*_lj_/*f*_c0_
	[%]	[MPa]	[MPa]	[-]	[-]	[-]
D15-P-M1-1L-1	0.296	4.00	340	9.24	0.250	0.109
D15-P-M1-1L-2	0.296	4.00	340	9.24	0.250	0.109
D15-P-M1-2L-1	0.593	8.01	682	18.474	0.501	0.217
D15-P-M2-2L-2	0.652	9.44	750	45.38	2.747	0.571
D15-P-M2-2L-3	0.652	9.44	750	21.63	0.624	0.272
D15-P-M2-2L-4	0.593	8.01	682	16.13	0.382	0.190
D15-P-M2-2L-5	0.652	9.44	750	14.22	0.270	0.179
D15-P-M2-2L-6	0.652	9.44	750	18.83	0.473	0.237
D15-P-M1-3L-1	0.889	12.02	1022	27.71	0.751	0.326
D20-P-M1-1L-1	0.222	3.00	256	9.48	0.352	0.111
D20-P-M3-1L-2	0.223	2.65	268	10.92	0.445	0.108
D20-P-M1-2L-1	0.444	6.00	511	18.96	0.703	0.223
D20-P-M3-2L-2	0.447	5.30	536	21.85	0.890	0.216
D20-P-M1-3L-1	0.733	10.62	843	31.28	1.160	0.394
D20-P-M3-3L-2	0.670	7.94	805	32.77	1.335	0.323
D25-P-M1-1L-1	0.178	2.40	204	7.28	0.259	0.086
D25-P-M1-2L-1	0.356	4.81	409	10.76	0.283	0.126
D25-P-M1-3L-1	0.533	7.21	613	16.14	0.425	0.190
D25-P-M1-4L-1	0.711	9.61	818	24.77	0.750	0.291
D30-P-M1-2L-1	0.296	4.00	341	11.06	0.359	0.130
D30-P-M1-3L-1	0.444	6.00	511	16.59	0.538	0.195

**Table 8:** Suggested approaches to determine *k*_ε_.

Source		FRP-Confined Plain Concrete	FRP-Confined Reinforced Concrete
Niedermeier	[33,40]	*k*_ε_ = 0.66, *k*_εk_ = 0.50	*k*_ε_ = 0.50, *k*_εk_ = 0.25
Lam and Teng	[6,23]	*k*_ε_ = 0.586 (Carbon), *k*_ε_ = 0.669 (Glass)	no information
Toutanji et al.	[41]	*k*_ε_ = 0.6	no information
Smith et al.	[21]	*k*_ε_ = 0.8	no information
Pellegrino and Modena	[8]	$k_{\varepsilon }=0.25+0.25\;\cdot \;\left(\frac{2\;\cdot {\;R}_{c}}{b}\right)$	$k_{\varepsilon }=\gamma \;\cdot {\;C}^{\;-0.7}\;\leq \;0.8\;\mathrm{with}\;C=\frac{E_{s}\;\cdot {\;\rho }_{l}}{E_{j\;}\cdot {\;\rho }_{j}}$

Abbreviations: *R*_c_ = corner radius; *E*_s_ = elastic modulus steel reinforcement; *ρ*_l_ = longitudinal steel ratio.

**Table 9:** Calculated partial factors *γ*_j_ for the CFRP materials used.

CFRP Sheet	*V*_x_	*γ*_j_
M1	0.200	1.59
M2	0.155	1.50
M3	0.189	1.57

**Table 10:** Recommended FRP material safety factors *γ*_j_.

Recommendation/Code		*γ*_j_
CNR-DT 200 R1/2013	[27]	1.21
GB 50608-2010	[28]	1.40
DAfStb-Guideline	[30]	1.35
fib Technical Report	[44]	1.35

**Table 11:** Test results of the CFRP-confined RC specimens.

Series	Specimens	*f*_c0_	*k*_e_	*f*_l(j+w)_	*f*_cc_	Δ*f*_cc_	*ε*_ccu_	*E*_2,t_	*ν*_2_
		[MPa]	[-]	[MPa]	[MPa]	[MPa]	[%]	[MPa]	[-]
D15-TR-M1-2L-1	1	42.3	0.352	9.93	83.80	36.70	1.254	5178	0.873
	2				89.46	42.36	1.680	5376	0.951
	3				86.15	39.05	1.720	4886	0.990
	***Mean:***				**86.47**	**39.37**	**1.551**	**5147**	**0.938**
D15-TR-M1-2L-2	1	42.3	0.182	8.51	83.25	36.16	1.620	3745	1.120
	2				81.92	34.82	1.430	3129	1.293
	3				73.03	25.94	1.180	4485	0.996
	***Mean:***				**79.40**	**32.31**	**1.410**	**3786**	**1.136**
D20-TR-M1-2L-1	1	27.0	0.154	6.08	65.08	27.08	1.980	3241	0.814
	2				69.37	31.37	2.176	2595	0.930
	3				67.76	29.76	2.106	2552	0.959
	***Mean:***				**67.40**	**29.40**	**2.087**	**2796**	**0.901**
D20-TR-M1-2L-2	1	27.0	0.146	6.17	64.99	26.99	1.977	3216	0.655
	2				64.43	26.43	1.915	2602	0.784
	3				60.75	22.75	1.746	2839	0.749
	***Mean:***				**63.93**	**25.39**	**1.879**	**2886**	**0.729**
D20-TR-M2-2L-3a	1	28.0	0.325	7.69	66.10	30.77	1.660	3945	0.647
	2				68.70	33.38	1.630	3476	0.736
	3				67.05	31.72	1.690	2860	0.971
	***Mean:***				**67.28**	**31.96**	**1.660**	**3427**	**0.785**
D20-TR-M2-2L-3b	1	28.0	0.325	7.69	72.80	33.75	1.690	3298	0.937
	2				75.91	36.85	1.860	3277	0.895
	3				72.84	33.78	1.660	3339	0.882
	***Mean:***				**73.85**	**34.79**	**1.737**	**3305**	**0.905**
D20-TR-M2-2L-3c	1	28.0	0.325	7.69	76.32	33.47	1.781	3631	0.811
	2				77.08	34.23	1.796	4370	0.769
	3				78.39	35.54	1.926	3524	0.781
	***Mean:***				**77.26**	**34.41**	**1.834**	**3842**	**0.787**
D20-TR-M2-2L-4	1	28.0	0.483	8.91	76.97	37.92	1.877	3738	0.727
	2				77.06	38.00	1.834	4424	0.654
	3				78.06	39.00	1.867	3973	0.709
	***Mean:***				**77.36**	**38.31**	**1.859**	**4045**	**0.697**
D20-TR-M2-1L-1	1	24.5	0.400	4.55	51.64	26.29	1.094	2830	0.880
	2				54.32	28.97	1.257	3190	0.865
	***Mean:***				**52.98**	**27.63**	**1.176**	**3010**	**0.873**
D20-TR-M2-1L-2	1	24.5	0.490	5.10	49.07	23.71	1.065	2452	0.941
	2				57.04	31.69	1.180	2043	1.228
	3				56.68	31.33	1.249	2303	1.072
	***Mean:***				**54.26**	**28.91**	**1.165**	**2266**	**1.080**
D20-TR-M2-1L-3	1	24.5	0.400	4.92	56.65	31.30	1.193	3871	0.783
	2				57.77	32.42	1.310	3129	0.921
	3				52.07	26.71	1.450	3621	0.891
	***Mean:***				**55.50**	**30.14**	**1.318**	**3540**	**0.865**
D25-SR-M1-1L-1	1	33.0	0.590	6.25	60.65	20.62	1.473	3125	0.799
	2				59.80	19.77	1.490	-	-
	3				60.84	20.81	1.616	3361	0.780
	***Mean:***				**60.43**	**20.40**	**1.526**	**3243**	**0.790**
D25-SR-M1-2L-1	1	39.0	0.590	8.65	76.51	30.50	1.850	3140	0.776
	2				75.79	29.78	1.966	3140	0.835
	3				76.69	30.68	2.036	3412	0.811
	***Mean:***				**76.33**	**30.32**	**1.951**	**3230**	**0.807**
D25-SR-M1-2L-2	1	28.1	0.578	10.75	-	-	-	5257	0.475
	2				-	-	-	4634	0.503
	3				-	-	-	4783	0.476
	***Mean:***				**-**	**-**	**-**	**4891**	**0.485**
D25-SR-M1-2L-3	1	31.2	0.590	8.65	68.08	29.86	1.911	3538	0.632
	2				68.96	30.74	2.214	4374	0.490
	***Mean:***				**68.52**	**30.30**	**2.063**	**3956**	**0.561**
D25-SR-M1-3L-1	1	39.0	0.590	11.06	87.95	41.94	2.350	4545	0.583
	2				87.25	41.24	2.220	4603	0.589
	3				85.88	39.87	2.100	4377	0.616
	***Mean:***				**87.03**	**41.02**	**2.223**	**4508**	**0.596**
D25-TR-M1-2L-1	1	33.0	0.430	5.43	60.90	20.86	1.800	2884	0.832
	2				57.57	17.54	1.605	2726	0.786
	3				50.83	10.80	1.258	2338	0.991
	***Mean:***				**56.43**	**16.40**	**1.554**	**2649**	**0.870**
D25-TR-M1-2L-2	1	31.2	0.430	5.43	54.02	15.80	1.466	2870	0.731
	2				50.83	12.61	1.289	2968	0.704
	3				54.64	16.42	1.564	2845	0.717
	***Mean:***				**53.16**	**14.94**	**1.440**	**2894**	**0.717**
D30-SR-M1-2L-1	1	31.0	0.651	7.44	-	-	-	4922	0.480
	2				-	-	-	4846	0.521
	3				-	-	-	4380	0.577
	***Mean:***				**-**	**-**	**-**	**4716**	**0.526**
D30-SR-M1-2L-2	1	31.0	0.601	7.63	-	-	-	4832	0.473
	2				65.20	29.34	1.880	3813	0.587
	3				-	-	-	3888	0.600
	***Mean:***				**65.20**	**29.34**	**1.880**	**4178**	**0.553**

**Table 12:** Specific values of the CFRP-confined RC specimens.

Series	*f*_lj_	*f*_l,wy_	*A*_sl_	*σ*_sl_	*f*_l(j+w)_/*f*_c0_
	[MPa]	[MPa]	[mm^2^]	[MPa]	[-]
D15-TR-M1-2L-1	8.01	1.92	170	4.85	0.235
D15-TR-M1-2L-2	8.01	0.50	170	4.85	0.201
D20-TR-M1-2L-1	6.01	0.07	679	11.04	0.226
D20-TR-M1-2L-2	6.01	0.16	679	11.04	0.229
D20-TR-M2-2L-3a	7.08	0.62	452	7.31	0.275
D20-TR-M2-2L-3b	7.08	0.62	679	11.04	0.275
D20-TR-M2-2L-3c	7.08	0.62	905	14.83	0.275
D20-TR-M2-2L-4	7.08	1.83	679	11.04	0.318
D20-TR-M2-1L-1	3.54	1.01	50	0.80	0.185
D20-TR-M2-1L-2	3.54	1.56	50	0.80	0.208
D20-TR-M2-1L-3	3.54	1.38	50	0.80	0.200
D25-SR-M1-1L-1	2.40	3.85	679	7.01	0.189
D25-SR-M1-2L-1	4.81	3.85	679	7.01	0.222
D25-SR-M1-2L-2	4.81	5.94	679	7.01	0.383
D25-SR-M1-2L-3	4.81	3.85	679	7.01	0.277
D25-SR-M1-3L-1	7.21	3.85	679	7.01	0.283
D25-TR-M1-2L-1	4.81	0.63	679	7.01	0.164
D25-TR-M1-2L-2	4.81	0.63	679	7.01	0.174
D30-SR-M1-2L-1	4.01	3.43	679	4.85	0.240
D30-SR-M1-2L-2	4.01	3.63	679	4.85	0.246

**Table 13:** Included experimental programs from the literature.

Authors			Used Materials	Number of Specimens ^1^
Xiao and Wu	(2003)	[13]	CFRP 1:	14 (U), 42 (U) *k*_1_ and *ν*_2_ analysis only
			*E*_j_ = 96 GPa, *ε*_FRP_ = 1.64 %, *t*_j,n=1_ = 0.39 mm	
			CFRP 2:	
			*E*_j_ = 78 GPa, *ε*_FRP_ = 1.59 %, *t*_j,n=1_ = 0.56 mm	
Lee et al.	(2004)	[46]	CFRP:	5 (U), 15 (R)
			*E*_j_ = 250 GPa, *ε*_FRP_ = 1.80 %, *t*_j,n=1_ = 0.11 mm	
			Spiral Reinforcement:	
			*f*_y_ = 1200 MPa, *D*_c_ = 130 mm	
			No Longitudinal Reinforcement	
Matthys et al.	(2005)	[47]	CFRP 1 (C240):	5 (R)
			*E*_j_ = 198 GPa, *ε*_FRP_ = 1.31 %	
			CFRP 2 (C640):	
			*E*_j_ = 480 GPa, *ε*_FRP_ = 0.23 %	
			GFRP (TU600/25):	
			*E*_j_ = 60 GPa, *ε*_FRP_ = 1.30 %	
			Hybrid (TU360G160C/27G):	
			*E*_j_ = 120 GPa, *ε*_FRP_ = 0.92 %	
			Transverse Reinforcement:	
			*f*_y_ = 560 MPa, *D*_c_ = 370 mm	
			Longitudinal Reinforcement:	
			*f*_y_ = 620 MPa, *n* = 10, *Ø* = 12 mm	
Lam et al.	(2004/2006)	[48,49]	CFRP (C):	18 (U)
			*E*_j_ = 230 GPa, *ε*_FRP_ = 1.49 %, *t*_j,n=1_ = 0.165 mm	
			GFRP (G):	
			*E*_j_ = 22 GPa, *ε*_FRP_ = 2.00 %, *t*_j,n=1_ = 1.27 mm	
Ilki et al.	(2008)	[50]	CFRP:	4 (R)
			*E*_j_ = 230 GPa, *ε*_FRP_ = 1.50 %, *t*_j,n=1_ = 0.165 mm	
			Transverse Reinforcement:	
			*f*_y_ = 476 MPa, *D*_c_ = 200 mm	
			Longitudinal Reinforcement:	
			*f*_y_ = 367 MPa, *n* = 6, *Ø* = 10 mm	
Eid et al.	(2009)	[4]	CFRP:	36 (U), 15 (R)
			*E*_j_ = 78 GPa, *ε*_FRP_ = 1.35 %, *t*_j,n=1_ = 0.38 mm	
			Transverse Reinforcement:	
			*f*_y_ = 456 MPa, *D*_c_ = 253 mm	
			Longitudinal Reinforcement:	
			*f*_y_ = 423 MPa, *n* = 6, *Ø* = 16 mm	

^1^ U, unreinforced specimens; R, reinforced specimens.

**Table 14:** Summarized results regarding the tests of the CFRP-confined plain concrete specimens.

Series	Specimens	*D*	*f*_c0_	*t*_j_	*f*_lj_	*f*_cc_	*ε*_ccu_	*E*_2,t_	*k*_ε_	*k*_1_	*ν*_2_
		[mm]	[MPa]	[mm]	[MPa]	[MPa]	[%]	[MPa]	[-]	[-]	[-]
Xiao and Wu (2003) [13]											
CFRP1-1L	1	152	33.7	0.39	4.68	48.0	1.35	1250	0.58	-	-
	2					50.0	1.24	1417	0.70	-	-
	3					50.0	1.40	1583	0.61	-	-
	***Mean:***					**49.3**	**1.33**	**1417**	**0.63**	**-**	**-**
CFRP1-2L	1	152	33.7	0.78	9.35	64.0	1.64	3167	0.55	-	-
	2					72.0	2.17	3300	0.61	-	-
	3					75.0	2.25	3750	0.61	-	-
	***Mean:***					**70.3**	**2.02**	**3406**	**0.59**	**-**	**-**
CFRP1-3L	1	152	33.7	1.17	14.03	83.0	2.48	5333	0.50	-	-
	2					87.0	2.45	6000	0.49	-	-
	3					95.5	3.00	6500	0.55	-	-
	***Mean:***					**88.5**	**2.64**	**5944**	**0.51**	**-**	**-**
CFRP2-1L	1	152	43.6	0.56	4.22	52.0	0.65	900	0.47	-	-
	2					54.5	0.78	1000	0.48	-	-
	3					-	-	-	-	-	-
	***Mean:***					**53.25**	**0.72**	**950**	**0.48**	**-**	**-**
CFRP2-1,5L	1	152	43.6	0.84	6.33	67.8	1.13	3150	0.45	-	-
	2					72.5	1.24	3350	0.41	-	-
	3					76.0	1.37	3760	0.50	-	-
	***Mean:***					**72.1**	**1.25**	**3420**	**0.45**	**-**	**-**
Lee et al. (2004) [46]											
S0F	1	150	36.2	0.11	4.05	41.7	1.00	517	0.64	1.41	-
	2			0.22	8.10	57.8	1.50	2381	0.51	3.25	0.67
	3			0.33	12.14	69.1	2.00	3311	0.55	3.01	0.47
	4			0.44	16.19	85.4	2.70	3854	0.69	2.63	0.54
	5			0.55	20.24	104.3	3.10	5477	0.67	2.99	0.38
Lam et al. (2004/2006) [48,49]											
C1	1	152	35.9	0.165	4.88	50.4	1.27	1375	0.65	2.75	0.91
	2					47.2	1.11	1375	0.67	2.75	1.09
	3					53.2	1.29	1813	0.77	3.63	0.83
	***Mean:***					**50.3**	**1.22**	**1521**	**0.70**	**3.04**	**0.94**
C2	1	152	35.9	0.330	9.76	68.7	1.68	3125	0.67	3.13	0.53
	2					69.9	1.96	3125	0.65	3.13	0.54
	3					71.6	1.85	3438	0.69	3.44	0.55
	***Mean:***					**70.1**	**1.83**	**3229**	**0.67**	**3.23**	**0.54**
C3	1	152	34.3	0.495	14.64	82.6	2.05	5625	0.54	3.75	0.38
	2					90.4	2.41	5363	0.61	3.58	0.42
	3					97.3	2.52	5938	0.66	3.96	0.40
	***Mean:***					**90.1**	**2.33**	**5642**	**0.60**	**3,76**	**0.40**
G1	1	152	38.5	1.27	6.36	56.2	-	-	-	-	-
	2					51.9	1.32	800	0.71	2.41	1.25
	3					58.3	1.46	900	0.96	2.13	1.33
	***Mean:***					**55.5**	**1.39**	**850**	**0.84**	**2.27**	**1.29**
G2	1	152	38.5	2.54	12.72	75.7	2.46	2000	0.83	2.66	0.95
	2					77.3	2.19	2227	0.88	2.97	0.89
	3					75.2	-	-	-	-	-
	***Mean:***					**76.1**	**2.32**	**2114**	**0.86**	**2.82**	**0.92**
CII-M	1	152	38.9	0.33	9.76	76.8	1.91	-	-	-	-
	2					79.1	2.08	-	-	-	-
	3					65.8	1.25	-	-	-	-
	***Mean:***					**73.9**	**1.75**	**-**	**-**	**-**	**-**
Eid et al. (2009) [4]											
N1	1	152	32.1	0.381	3.83	39.0	1.00	1000	0.60	2.56	0.80
	2					41.0	1.08	1083	0.62	2.77	0.92
	3					41.0	1.08	1083	0.62	2.77	0.92
	***Mean:***					**40.3**	**1.05**	**1055**	**0.61**	**2.70**	**0.88**
N2	1	152	32.1	0.762	7.65	58.0	2.00	2617	0.74	3.35	0.48
	2					57.5	1.79	2500	0.67	3.20	0.50
	3					57.5	1.79	2583	0.69	3.30	0.51
	***Mean:***					**57.7**	**1.86**	**2567**	**0.70**	**3.28**	**0.50**
N3	1	152	33.6	1.143	11.48	72.5	2.23	4333	0.63	3.69	0.39
	2					75.0	2.32	4417	0.65	3.77	0.40
	3					77.0	2.43	4583	0.65	3.91	0.40
	***Mean:***					**74.8**	**2.33**	**4444**	**0.64**	**3.79**	**0.40**
M1	1	152	48.0	0.381	3.83	57.0	0.62	500	0.58	1.28	-
	2					60.5	0.66	500	0.66	1.28	1.75
	3					62.0	0.78	700	0.63	1.79	1.79
	***Mean:***					**59.8**	**0.69**	**567**	**0.62**	**1.45**	**1.77**
M2	1	152	48.0	0.762	7.65	79.5	1.23	2050	0.82	2.62	1.10
	2					79.5	1.23	2050	0.82	2.62	1.14
	3					81.0	1.18	2500	0.98	3.20	1.03
	***Mean:***					**80.0**	**1.21**	**2200**	**0.87**	**2.81**	**1.09**
M3	1	152	48.0	1.143	11.48	97.0	1.48	3200	0.88	2.73	0.94
	2					101.0	1.60	3200	1.06	2.73	1.04
	3					102.0	1.70	3200	1.06	2.73	1.07
	***Mean:***					**100.0**	**1.59**	**3200**	**1.00**	**2.73**	**1.02**
H11	1	152	67.7	0.381	3.83	57.5	0.63	-	0.59	-	-
	2					61.5	0.67	-	0.73	-	-
	3					66.0	0.69	-	0.77	-	-
	***Mean:***					**61.7**	**0.66**	**-**	**0.70**	**-**	**-**
H12	1	152	67.7	0.762	7.65	72.5	0.89	-	0.71	-	-
	2					83.0	1.08	417	0.91	0.53	1.90
	3					84.0	1.14	667	1.00	0.85	1.44
	***Mean:***					**79.8**	**1.04**	**542**	**0.87**	**0.69**	**1.67**
H13	1	152	75.9	1.143	11.48	89.0	1.01	-	0.87	-	-
	2					97.0	1.08	750	0.74	0.64	1.56
	3					97.0	1.20	1083	0.89	0.92	1.19
	***Mean:***					**94.3**	**1.10**	**917**	**0.83**	**0.78**	**1.38**
H21	1	152	107.7	0.381	3.83	91.0	0.52	-	0.56	-	-
	2					91.0	0.52	-	0.56	-	-
	3					92.5	0.54	-	0.53	-	-
	***Mean:***					**91.5**	**0.53**	**-**	**0.55**	**-**	**-**
H22	1	152	107.7	0.762	7.65	88.0	0.85	-	0.81	-	-
	2					95.5	0.73	-	0.56	-	-
	3					105.5	0.79	-	0.67	-	-
	***Mean:***					**96.3**	**0.79**	**-**	**0.68**	**-**	**-**
H23	1	152	107.7	1.143	11.48	105.0	1.00	-	0.74	-	-
	2					112.5	0.71	-	0.53	-	-
	3					117.0	0.88	-	0.65	-	-
	***Mean:***					**111.5**	**0.86**	**-**	**0.64**	**-**	**-**

**Table 15:** Summarized results regarding the tests of the CFRP-confined RC specimens.

Series	*D*	*f*_c0_	*t*_j_	*f*_lj_	*s*	*Ø*_w_	*k*_e_	*f*_l,wy_	*f*_cc_	*ε*_ccu_
	[mm]	[MPa]	[mm]	[MPa]	[mm]	[mm]	[-]	[MPa]	[MPa]	[%]
Lee et al. (2004) [46]										
S6F1	150	36.2	0.110	4.05	60	5.5	0.44	3.25	50.37	1.70
S6F2	150	36.2	0.220	8.10	60	5.5	0.44	3.25	68.52	2.50
S6F4	150	36.2	0.440	16.19	60	5.5	0.44	3.25	99.49	3.40
S6F5	150	36.2	0.550	20.24	60	5.5	0.44	3.25	114.64	3.60
S4F1	150	36.2	0.110	4.05	40	5.5	0.54	5.90	60.00	1.90
S4F2	150	36.2	0.220	8.10	40	5.5	0.54	5.90	74.77	2.30
S4F3	150	36.2	0.330	12.14	40	5.5	0.54	5.90	73.85	2.90
S4F4	150	36.2	0.440	16.19	40	5.5	0.54	5.90	104.15	3.00
S4F5	150	36.2	0.550	20.24	40	5.5	0.54	5.90	123.64	3.60
S2F1	150	36.2	0.110	4.05	20	5.5	0.64	14.04	72.87	2.20
S2F2	150	36.2	0.220	8.10	20	5.5	0.64	14.04	92.68	3.60
S2F3	150	36.2	0.330	12.14	20	5.5	0.64	14.04	108.01	3.90
S2F4	150	36.2	0.440	16.19	20	5.5	0.64	14.04	115.72	3.80
S2F5	150	36.2	0.550	20.24	20	5.5	0.64	14.04	150.80	4.30
Matthys et al. (2005) [47]										
K2	400	34.3	0.585	4.64	140	8	0.53	0.59	59.36	1.20
K3	400	34.3	0.940	5.89	140	8	0.53	0.59	59.60	0.43
K4	400	39.3	1.800	4.21	140	8	0.53	0.59	60.32	0.69
K5	400	39.3	0.600	1.40	140	8	0.53	0.59	42.38	0.38
K8	400	39.1	0.492	1.49	140	8	0.53	0.59	49.58	0.60
Ilki et al. (2008) [50]										
NSR-C-050-3	250	27.6	0.495	9.51	50	8	0.45	2.22	77.59	3.40
NSR-C-100-3	250	27.6	0.495	9.51	100	8	0.32	0.80	72.60	2.80
NSR-C-145-3	250	27.6	0.495	9.51	145	8	0.23	0.39	71.95	3.30
NSR-C-145-5	250	27.6	0.825	15.85	145	8	0.23	0.39	94.45	4.50
Eid et al. (2009) [4]										
A5NP2C	303	29.4	0.762	3.84	150	9.5	0.31	0.72	46.13	0.63
A3NP2C	303	31.7	0.762	3.84	70	9.5	0.47	2.37	60.06	1.24
A1NP2C	303	31.7	0.762	3.84	45	9.5	0.53	4.14	63.39	1.51
C4NP2C	303	31.7	0.762	3.84	100	11.3	0.40	1.51	51.37	0.77
C4N1P2C	303	36.0	0.762	3.84	100	11.3	0.40	1.51	56.87	0.84
C4NP4C	303	31.7	1.524	7.68	100	11.3	0.40	1.51	75.83	2.08
B4NP2C	303	31.7	0.762	3.84	100	11.3	0.40	1.51	58.00	1.36
C4MP2C	303	50.8	0.762	3.84	100	11.3	0.40	1.51	75.36	0.88
C2NP2C	303	31.7	0.762	3.84	65	11.3	0.48	2.78	55.94	1.32
C2N1P2C	303	36.0	0.762	3.84	65	11.3	0.48	2.78	62.44	1.03
C2N1P4C	303	36.0	1.524	7.68	65	11.3	0.48	2.78	75.71	1.84
C2N1P2N	303	36.0	0.762	4.60	65	11.3	0.68	3.98	75.57	1.55
C2MP2C	303	50.8	0.762	3.84	65	11.3	0.48	2.78	78.90	1.04
C2MP4C	303	50.8	1.524	7.68	65	11.3	0.48	2.78	97.94	1.64
C2MP2N	303	50.8	0.762	4.60	65	11.3	0.68	3.98	62.45	1.29

**Table 16:** Additional data concerning *ν*_2_ and *k*_1_.

*E*_jl_/*f*_c0_	*ν*_2_	*E*_jl_/*f*_c0_^2^	*k*_1_
[-]	[-]	[-]	[-]
47.00	0.30	1.30	3.80
47.00	0.35	1.30	4.00
47.00	0.35	1.30	4.40
36.00	0.40	0.88	3.20
36.00	0.42	0.88	3.40
36.00	0.45	0.88	4.00
31.00	0.41	0.80	3.35
31.00	0.42	0.80	3.75
31.00	0.49	0.80	4.20
28.50	0.55	0.78	3.25
28.50	0.61	0.78	3.80
28.50	0.61	0.78	3.80
26.50	0.39	0.53	3.20
26.50	0.44	0.53	3.50
26.50	0.60	0.53	3.35
24.00	0.55	0.50	2.70
24.00	0.61	0.50	3.00
24.00	0.73	0.50	3.20
19.50	0.55	0.48	3.25
19.50	0.60	0.48	3.25
19.50	0.60	0.48	3.40
17.50	0.58	0.43	3.70
17.50	0.65	0.43	3.90
17.50	0.73	0.43	4.20
16.00	1.25	0.42	2.55
16.00	1.30	0.42	2.75
16.00	1.68	0.42	3.05
15.50	0.75	0.31	0.45
15.50	0.80	0.31	0.70
15.50	0.85	0.31	1.00
13.00	1.34	0.30	0.45
13.00	1.71	0.30	1.20
13.00	1.85	0.30	2.20
10.50	1.45	0.28	-0.95
10.50	1.82	0.28	0.05
8.50	1.10	0.28	1.75
8.50	1.42	0.25	0.30
6.00	1.45	0.25	0.75
6.00	2.09	0.25	0.90
6.00	2.45	0.16	-4.30
-	-	0.16	-1.00
-	-	0.16	0.65
